# Synthesis and Structural Analysis of New (−)-Cytisine Squaramides

**DOI:** 10.3390/molecules30051135

**Published:** 2025-03-01

**Authors:** Anna K. Przybył, Jan Janczak, Adam Huczyński

**Affiliations:** 1 Department of Medical Chemistry, Faculty of Chemistry, Adam Mickiewicz University, Uniwersytetu Poznańskiego 8, 61-614 Poznań, Poland; 2 Institute of Low Temperature and Structure Research, Polish Academy of Sciences, Okólna 2 Str., 50-422 Wrocław, Poland; j.janczak@intibs.pl

**Keywords:** squaric acid, cytisine, squaramide, crystal structure, derivatives

## Abstract

Derivatives of squaric acid are valuable building blocks with promising applications in the investigation of various bioactivities. In this study, we focus on squaramides functionalized with the (−)-cytisine moiety, an alkaloid known for its bioactivity as a nicotinic acetylcholine receptor agonist and its application in nicotine addiction treatment. Reactions of cytisine-monosquarate with several amines, such as ammonia, propargylamine, and morpholine, led to the formation of novel conjugates of cytisine-squaramides. Additionally, squaramide containing two cytisine moieties was synthesized via the reaction of diethyl squarate with cytisine at a 1:2 molar ratio. All obtained squaramides were thoroughly characterized by MS, FT-IR, and NMR methods and by single-crystal X-ray diffraction analysis. To gain deeper insights into their structural properties and intermolecular interactions, geometry optimizations were performed using DFT calculations, complemented with 3D molecular electrostatic potential maps.

## 1. Introduction

**Scheme 1:**
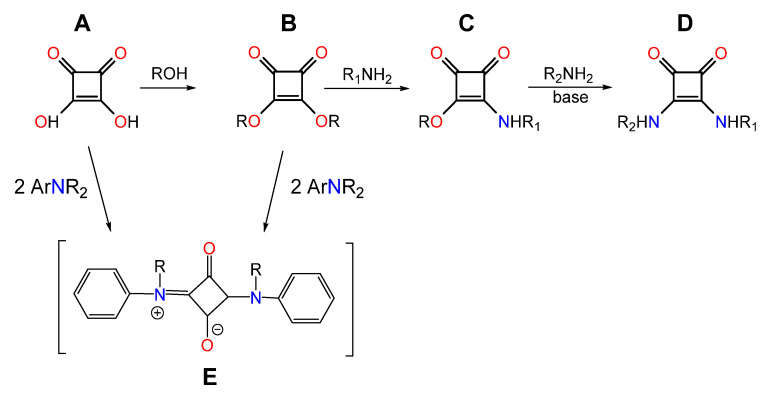
Squaric acid derivatives: (**A**) squaric acid, (**B**) squarate ester, (**C**) monosquarate-amide, (**D**) squaramide [2,5,6], (**E**) squaraines dye [7,8].

Squaric acid (**A**, Scheme 1) has gained considerable interest in chemistry, materials science, and pharmaceuticals due to its unique and versatile properties [1]. Unlike typical carboxylic acids, squaric acid does not have a carboxyl group, yet its pronounced acidity stems from the inductive effects of oxygen atoms, the presence of a double bond, and most importantly, from the resonance stabilization of its ionic forms. In its dianionic state, the negative charges are evenly delocalized across all oxygen atoms, further enhancing its stability [2]. Squaric acid is characterized by its ability to form strong and stable hydrogen bonds thanks to two acidic hydroxyl groups and two highly polar carbonyl groups. The high acidity of squaric acid is reflected in its dissociation constants, with values of pKa_1_ = 0.54 and pKa_2_ = 3 [2,3].

In recent years, there has been a significant interest in synthesizing derivatives of squaric acid due to their wide-ranging applications. Mono-squaramides (**C**) squaramides (**D**) and other derivatives **B** and **E** (Scheme 1) are promising building blocks in medicinal chemistry, opening new avenues for drug discovery and therapeutic innovations. Specifically, squaric acid and its derivatives exhibit a range of bioactivities, including antiparasitic, antibacterial, cytotoxic and antiviral properties, making these compounds valuable candidates for therapeutic applications. In addition, some derivatives have also demonstrated potential as effective drug carriers [2]. Among the latter, squaramides stand out for their synthesis via reactions of squaric acid with antimalarial drugs such as clindamycin, chloroquine, and mortiamide D. The resulting conjugates have shown enhanced pharmacological activity compared to the parent drugs, underscoring their potential in modern drug development [4].

Prototypes of vaccines based on squaric acid (**A**) chemistry have been found promising in combating dysentery caused by bacteria *Shigella bacilli*, as demonstrated in mouse studies. Squaric acid has also been explored in the development of vaccines against cholera [9]. In cancer treatment, squaric acid drug conjugates are particularly valued for their rapid and straightforward synthesis. Moreover, these compounds exhibit no negative effects when used for radiopharmaceuticals labeling, making them an excellent choice for diagnostic and therapeutic applications [10,11,12].

Squaric acid has been identified as a selective ionophore for detection of non-steroidal anti-inflammatory drugs (NSAIDs), such as ketoprofen and naproxen, in aqueous environments. Given the toxicity of the latter compounds at high concentrations, their constant monitoring is essential and the conjugates of squaric acid obtained with the above-mentioned drugs have been successfully detected using potentiometric methods [13]. In addition to detection capabilities, squaramides may contribute to environmental remediation.

Polysquaramide-functionalized carbon nanoparticles have been developed as a novel adsorbent, demonstrating the ability to purify water by effectively removing heavy metals, particularly mercury [14]. These remarkable properties underscore the broad utility of squaramides in various domains. In medicinal chemistry, they have already found applications as drugs, drug carriers, and model compounds in the development of active photosensitizers, inhibitors, potassium channel openers, anticancer agents, and antihistaminic compounds [15].

Furthermore, squaramide derivatives have been explored for their impact on the central nervous system (CNS). Dipeptide derivatives bearing the bis-squaramide backbone have shown promise in inhibiting amyloid β (Aβ) synthesis, offering potential therapeutic avenues for Alzheimer’s disease and other dementia-related conditions over the long-term administration [16].

**Figure 1:**
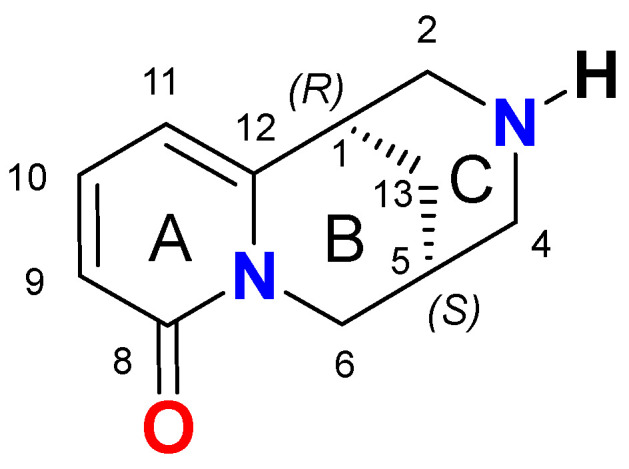
Structure of (−)-cytisine (**1**).

This line of research aligns closely with studies on (−)-cytisine (**1**, Figure 1), a bioactive alkaloid extensively used as a model compound in CNS research [17,18,19,20,21]. Previous findings have highlighted (−)-cytisine’s ability to coordinate zinc and problematic copper ions, suggesting its potential in addressing metal ion imbalances often implicated in neurodegenerative diseases [22]. By integrating the properties of squaric acid and (−)-cytisine through the synthesis of monosquarate-cytisine or bisquaramide derivatives, novel model compounds could be developed. Such novel compounds may advance the study of amyloid β (Aβ) inhibition or serve as tools for copper ion coordination in complex biological environments.

Moreover, (−)-cytisine (**1**, Figure 1), as an effective agonist of nicotinic acetylcholine receptors, has already found widespread use in smoking cessation therapy. As its therapeutic applications expand, the need for accurate dose control has become apparent. Recent advancements propose the use of luminescence detection methods paired with smartphone applications to enable accessible and visual cytisine monitoring, enhancing the practicality of home therapies [23].

## 2. Results and Discussion

### 2.1. Chemistry

**Scheme 2:**
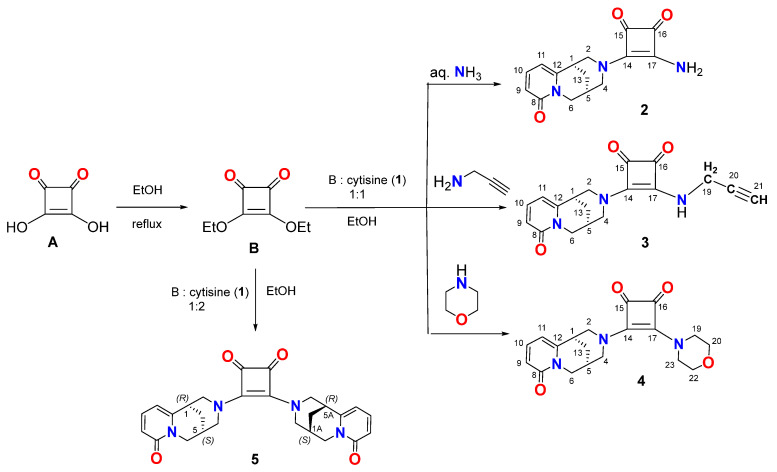
Synthesis of the bis-squaramides **2**–**5** from the diethylsquarate (**1**).

In order to develop new cytisine derivatives, we have designed a simple synthesis of (−)-cytisine conjugated with squaric acid and subsequent modifications of the cytisine containing monosquaramide to obtain new bifunctional squaramides (Scheme 2).

For this purpose, (−)-cytisine was refluxed with diethylsquarate at a 1:1 ratio for 6 h to obtain cytisine monosquaramide. In the next step, the monosquaramide was treated with various amines: ammonia, propargylamine, morpholine, and additionally with (−)-cytisine, to obtain bis-squaramides **2**, **3**, **4**, and **5**, respectively (Scheme 2). After work-up and purification using flash chromatography, the novel compounds were crystallized and analyzed using spectroscopic methods and single-crystal X-ray diffraction.

### 2.2. X-Ray Structure Characterization

#### 2.2.1. (−)-Cytisine Squaramide **2**

**Figure 2:**
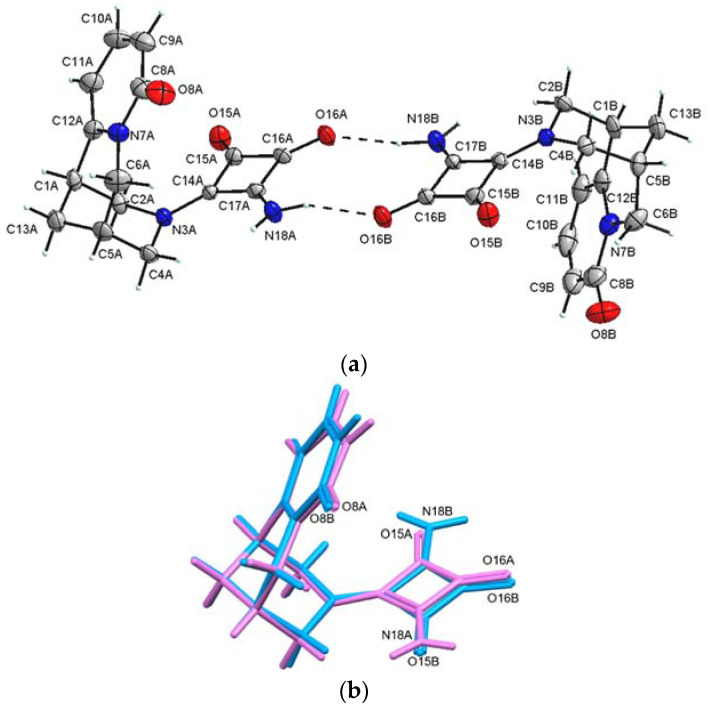
(**a**) View of the asymmetric unit of compound **2** and (**b**) overlay of both independent molecules (pink—Mol_A, blue—Mol_B).

(−)-Cytisine squaramide **2** crystallizes in the non-centrosymmetric space group of the triclinic system with two molecules per unit cell (Figure 2). The two (−)-cytisine squaramide molecules are independent and interact with each other through a pair of N–H⋯O hydrogen bonds formed between the amine groups and carbonyl oxygen atoms as shown in Figure 2a. The conformation of the two independent molecules is only slightly different, as shown in Figure 2b. However, the main difference is observed in the NH_2_ groups in these independent molecules. The squaramide group in molecule B is rotated by ~180° around the N3–C14 bond relative to its orientation in molecule A. This arrangement is stabilized by the N–H⋯O interactions between molecule A and molecule B (Table 1, Figure 2).

**Figure 3:**
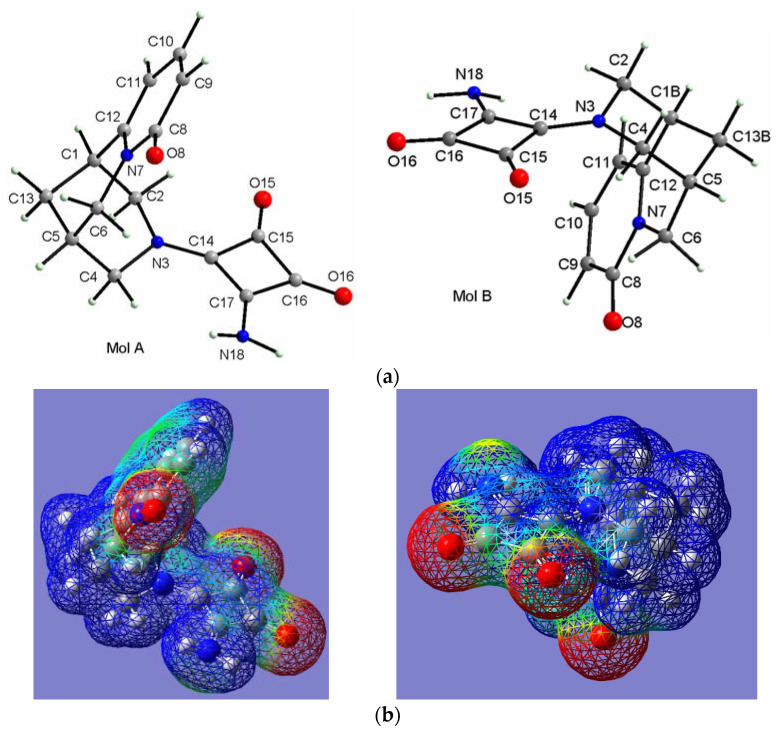
(**a**) View of the DFT-optimized conformation of (−)-cytisine squaramide **2** molecules and (**b**) their 3D molecular electrostatic potential mapped onto the total electron density isosurface (0.008 eÅ^−3^). Red and blue in the EP represent values between −0.05 and 0.05 eÅ^−1^. (Molecule A–left and Molecule B–right).

The fully DFT-optimized structures of (−)-cytisine squaramide **2**, obtained separately for both independent molecules A and B, showed that the conformation of molecule A is only slightly more stable than that of molecule B by approximately 3.074 kJ/mol. Both DFT-optimized geometries are very similar to those observed in the crystal (Figure 3).

Selected conformational parameters are listed together with the X-ray data in Table 2, whereas the parameters of fully DFT-optimized structures of molecules A and B are listed in Table S1a,b (in Supplementary Materials). The opposite orientation of the squaramide group in relation to (−)-cytisine in molecules A and B is clearly manifested by the rotation angle of C2–N3–C14–C15 (Table 2).

**Figure 4:**
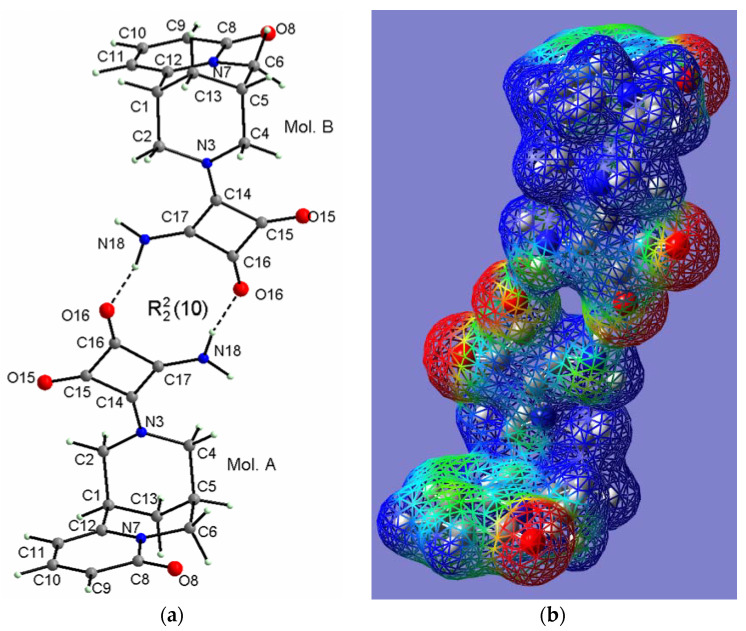
(**a**) View of the DFT-optimized conformation of (−)-cytisine squaramide **2** dimeric structure with a graph of $R_{2}^{2}\left(10\right)$ and (**b**) 3D molecular electrostatic potential mapped onto the total electron density isosurface (0.008 eÅ^−3^). Red and blue in the EP represent values between −0.05 and 0.05 eÅ^−1^.

In crystal **2**, the (−)-cytisine squaramide molecules A and B in the asymmetric unit interact with each other through a pair of N–H⋯O hydrogen bonds, forming a dimeric structure with a graph of $R_{2}^{2}\left(10\right).$ The formation of a dimeric structure with a graph of $R_{2}^{2}\left(10\right)$ takes place between the fragments of molecules with different signs of electrostatic potential (Figure 3b). Since molecules A and B form dimeric structures in the crystal, the geometry optimization for such dimers was performed using the DFT methods. The optimized conformation of such an AB dimer is shown in Figure 4, while detailed geometrical parameters are listed in Table S1c (in Supplementary Materials). The energy stabilization of such a dimeric structure (AB) is 16.19 kcal/mol (67.75 kJ/mol), which is a measure of the strength of the pair of N–H⋯O hydrogen bonds, with a graph of $R_{2}^{2}\left(10\right)$ forming the dimer, compensating for the slight energy increase in Mol B, and stabilizing the AB dimer. The 3D molecular electrostatic potential mapped onto the total electron density isosurface (0.008 eÅ^−3^) for the AB dimer is shown in Figure 4b, which is helpful for elucidating the interactions between the AB dimers.

**Figure 5:**
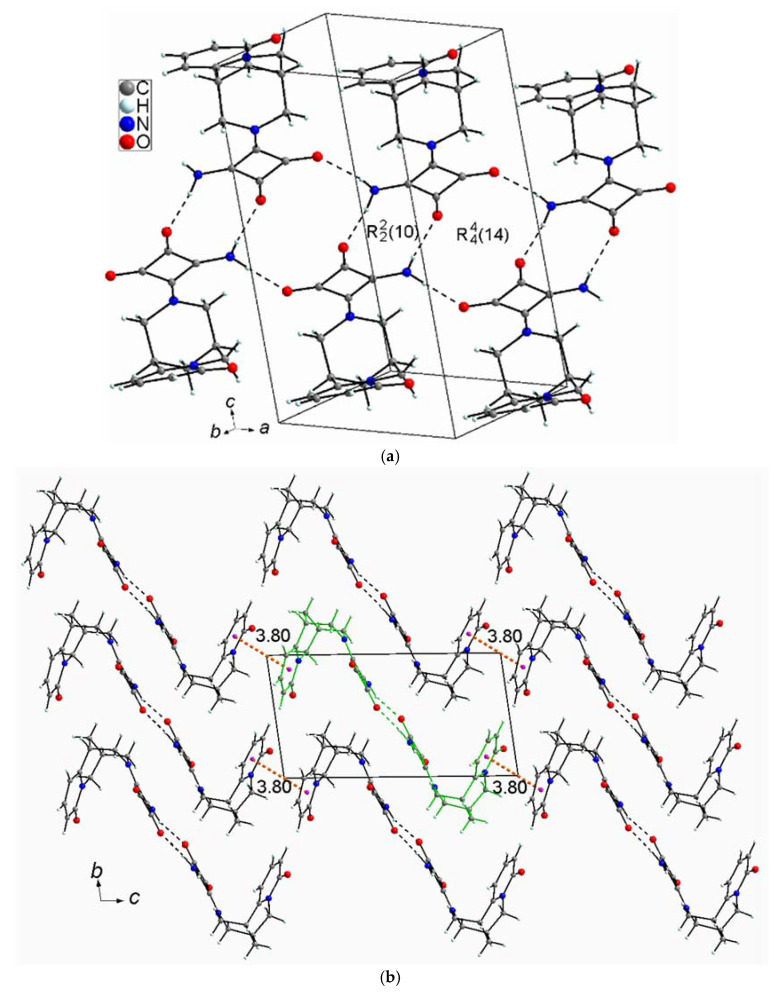
(**a**) Chain of (−)-cytisine squaramide molecules in crystal **2** and (**b**) arrangement of chains stabilized by a π⋯π interaction in the crystal seen along the *a*-axis. One of the chains is marked in green.

Translationally related dimers are linked with each other along the *a*-axis using a pair of N–H⋯O hydrogen bonds with a graph of $R_{4}^{4}\left(14\right)$ to form a chain (Figure 5a). The chains are translationally related along the *c* and *b* axes and are stabilized by weak stacking interactions between the pyridine rings. The distance of ~3.80 Å between the centers of gravity of the pyridine rings in the stacks indicates a weak π⋯π interaction stabilizing the crystal structure (Figure 5b).

#### 2.2.2. (−)-Cytisine Squaramide **3**‧H_2_O

**Figure 6:**
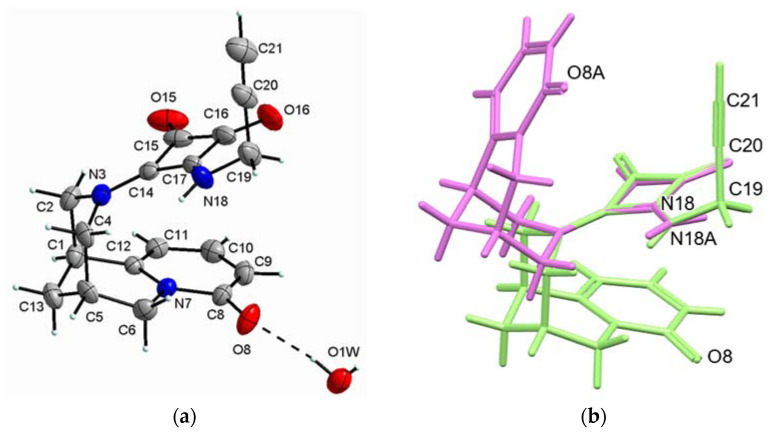
View of the asymmetric unit of **3‧**H_2_O (**a**) and its overlay with Mol_A of **2** (**b**). Water molecule is omitted (green—molecule **3** and pink—Mol_A of **2**).

(−)-Cytisine squaramide **3** crystallizes in the non-centrosymmetric space group P2_1_2_1_2_1_ of orthorhombic system as monohydrate (**3‧**H_2_O) with four molecules per unit cell. The asymmetric unit of **3‧**H_2_O contains one molecule of (−)-cytisine squaramide and one water molecule joined together by the O–H⋯O hydrogen bond, in which the water molecule acts as a donor (Figure 6a). Substitution of one hydrogen atom in the amino group of the square acid ring by a rather long 2-propynyl group causes, due to the steric hindrance, a change in the chair conformation into another chair conformation of the piperidine ring connected to the square ring, as shown in Figure 6b by overlaying molecule **3** (marked in green) with one (Mol_A) of the molecules **2** (marked in pink).

**Figure 7:**
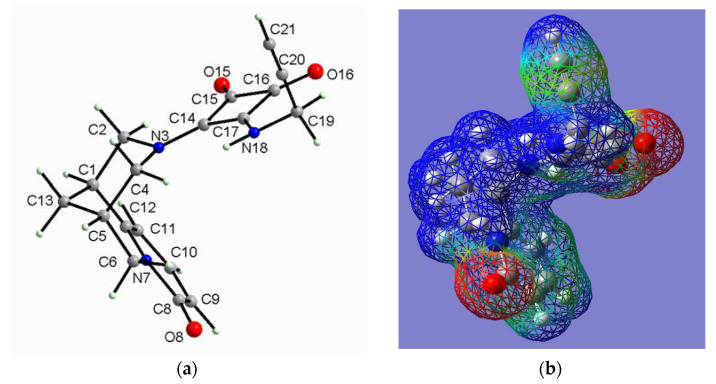
(**a**) View of the DFT-optimized conformation of (−)-cytisine squaramide **3** and (**b**) its 3D molecular electrostatic potential mapped onto the total electron density isosurface (0.008 eÅ^−3^). Red and blue in the EP represent values between −0.05 and 0.05 eÅ^−1^.

The fully DFT-optimized structure of (−)-cytisine squaramide **3** was also determined and showed a fairly similar conformation to that of the crystal (Figure 7). After optimization of the geometry of cytisine squaramide **3**, the molecular electrostatic potential was also calculated, which is useful for the organization of molecules and the interactions between them during nucleation and crystal growth (Figure 7b).

Selected conformational parameters are listed together with the X-ray data in Table 3, whereas the fully DFT-optimized parameters are listed in Table S2 (in Supplementary Materials). The main difference between the X-ray and DFT conformation of cytisine squaramide **3** is observed in the orientation of the squaramide ring in relation to cytisine molecule, which is clearly manifested in the rotation angles of C1–C2–N3–C14 and C2–N3–C14–C15 (Table 3).

**Figure 8:**
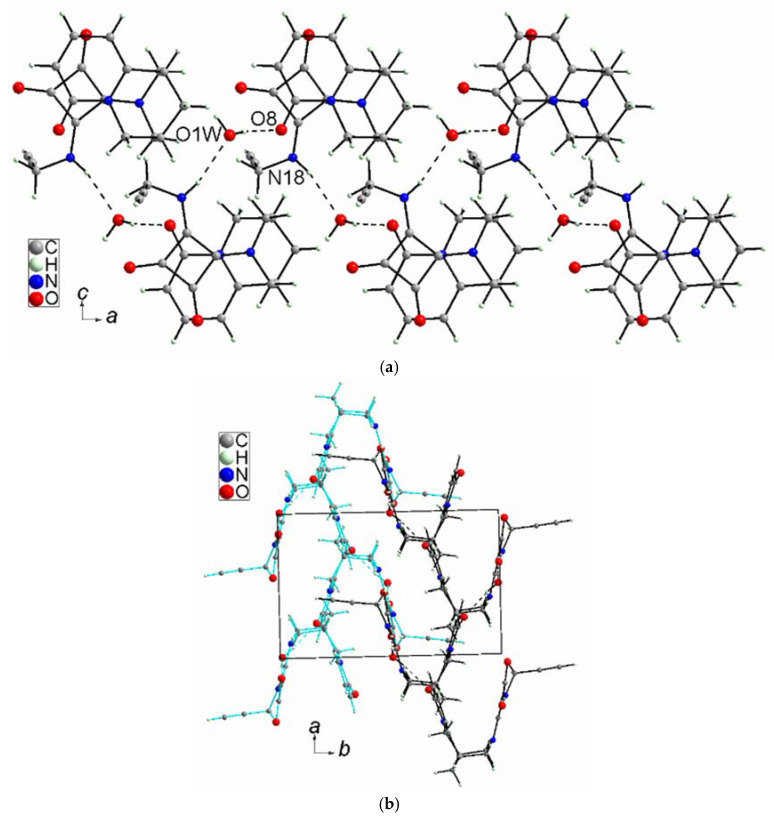
(**a**) Hydrogen-bonded ribbon of molecules **3‧**H_2_O running along the *a*-axis and (**b**) the packing diagram of **3‧**H_2_O viewed along the *c*-axis showing two adjacent and interpenetrating hydrogen bonded ribbons; one of the tapes is marked in blue.

The molecules of (−)-cytisine squaramide **3** related by a twofold screw axis and translation along the *a*-axis are connected by water molecules via N–H⋯O and O–H⋯O hydrogen bonds, forming ribbons in the direction of the *a*-axis as shown in Figure 8a. In these ribbons, the water molecules act as donors and as acceptors in the hydrogen bonds (Table 4).

There are no directional interactions such as hydrogen bonds between the ribbons in the crystal. However, neighboring ribbons are interdigitated by their 2-propynyl groups that are oriented almost parallel to the *b*-axis as shown in Figure 8b.

#### 2.2.3. (−)-Cytisine Squaramide **4**

**Figure 9:**
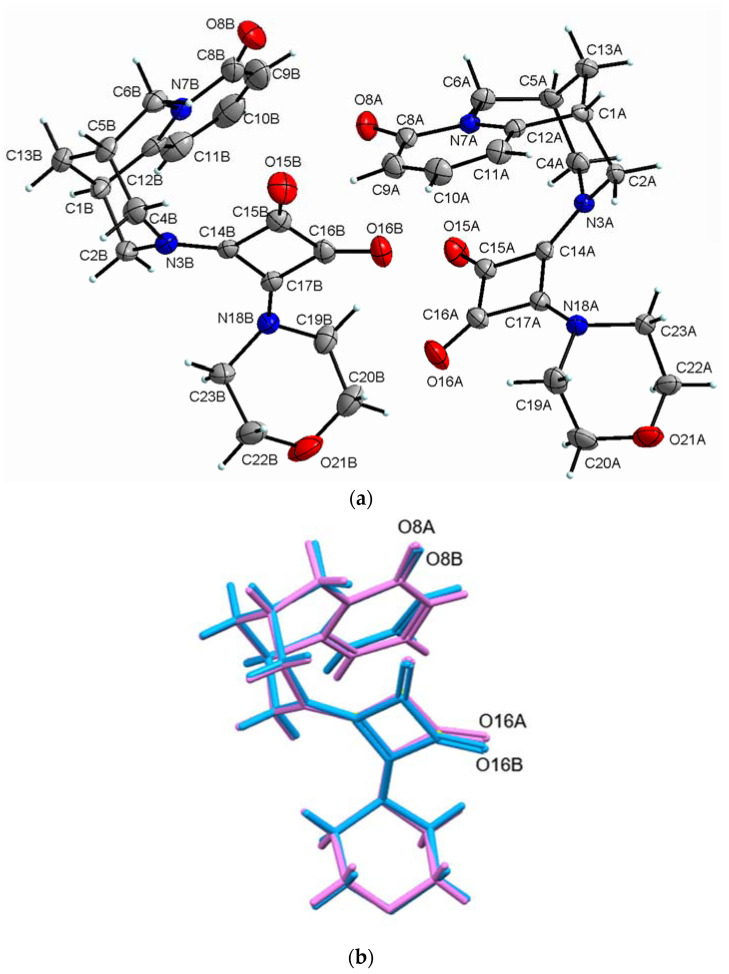
(**a**) View of the asymmetric unit of compound **4** and (**b**) overlay of both molecules (pink—Mol_A, blue—Mol_B).

The (−)-cytisine squaramide **4** crystallizes in the non-centrosymmetric space group P2_1_ of the monoclinic system with four molecules per unit cell, but the asymmetric unit contains two molecules (Figure 9a). The conformations of both independent cytisine squaramide **4** molecules are very similar, as shown in Figure 9b.

However, the most pronounced difference between the conformations is in the tilt of the pyridine ring to the square ring. The dihedral angle between the planes of these rings in molecule A is slightly smaller (~15.6(2)°) than in molecule B (~26.4(2)°).

**Figure 10:**
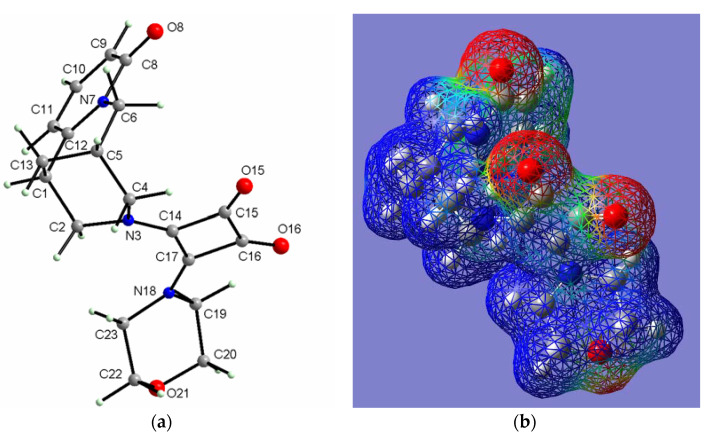
(**a**) View of the DFT-optimized conformation of (−)-cytisine squaramide **4** and (**b**) its 3D molecular electrostatic potential mapped onto the total electron density isosurface (0.008 eÅ^−3^). Red and blue in the EP represent values between −0.05 and 0.05 eÅ^−1^.

The fully optimized DFT structure of cytisine squaramide **4** obtained separately for both independent molecules A and B showed identical conformation, quite similar to that in the crystal (Figure 10). Selected conformational parameters are listed together with the X-ray data in Table 5, whereas the fully DFT-optimized parameters are listed in Table S3 (in Supplementary Materials).

**Figure 11:**
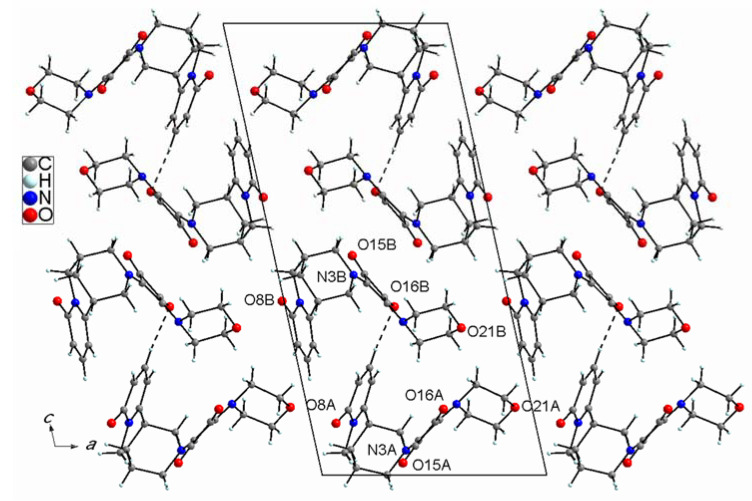
Packing diagram of **4** showing weak C–H⋯O contacts between molecules A and B.

The arrangement of cytisine squaramide **4** molecules in the crystal is mainly determined by van der Waals forces, such as dispersion forces and electrostatic interactions between molecular fragments characterized by the opposite EP signs, since there are no typical directional interactions, e.g., hydrogen bonds (Figure 11). At most, we can observe a weak C–H⋯O contact with the distance H⋯O = 2.41 Å and the angle C–H⋯O = 163° between molecules A and B in the asymmetric unit, while between the asymmetric units this type of contacts are slightly longer.

#### 2.2.4. (−)-Cytisine Squaramide **5**

**Figure 12:**
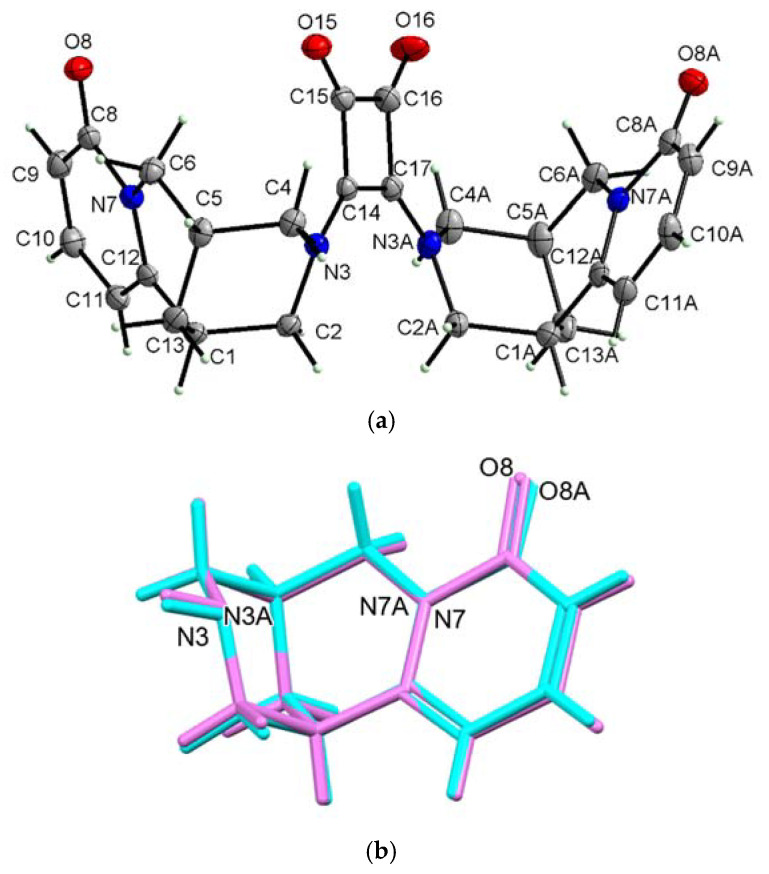
View of the asymmetric unit of **5** (**a**) and the overlay of the conformation of both (−)-cytisine moieties linked to the square acid ring (**b**).

The (−)-cytisine squaramide **5** crystallizes in the non-centrosymmetric space group P2_1_ of the monoclinic system with two molecules per unit cell. The asymmetric unit of **5** consists of one molecule (Figure 12). The conformation of both cytisine moieties linked to the square acid ring at C14 and C17 are almost the same, as shown in Figure 12b.

**Figure 13:**
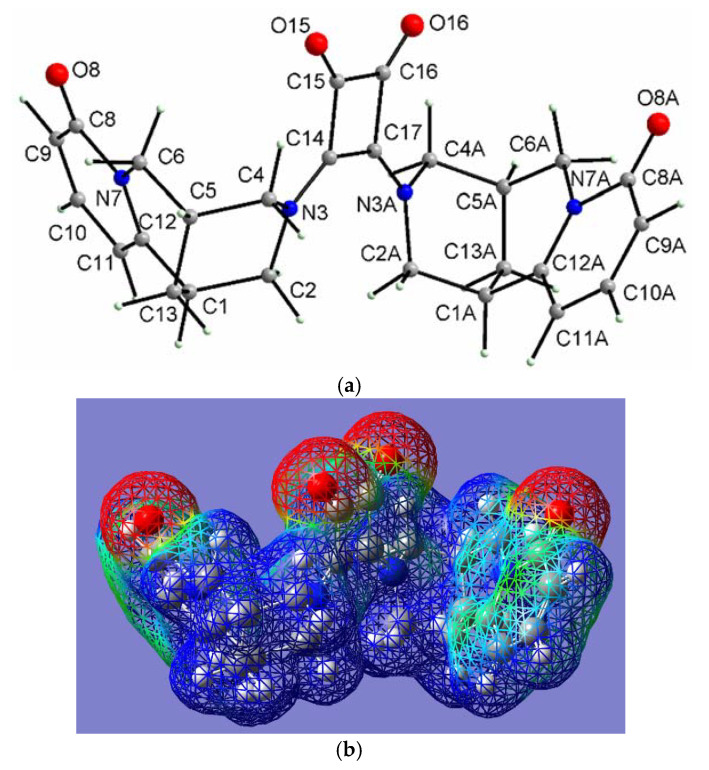
(**a**) View of the DFT-optimized conformation of (−)-cytisine squaramide **5** and (**b**) its 3D molecular electrostatic potential mapped onto the total electron density isosurface (0.008 eÅ^−3^). Red and blue in the EP represent values between −0.05 and 0.05 eÅ^−1^.

The fully DFT-optimized structure of (−)-cytisine squaramide **5** was also determined. The optimized conformation of cytisine squaramide **5** is fairly similar to that of the crystal (Figure 13a). After optimization of the geometry of cytisine squaramide **5**, the molecular electrostatic potential was also calculated, which is useful in explaining the organization of molecules and the interactions between them during nucleation and crystal growth (Figure 13b).

Selected conformational parameters are listed together with the X-ray values in Table 6, whereas the fully DFT-optimized parameters are listed in Table S4 (in Supplementary Materials). Although no symmetry was imposed during the DFT calculations, the optimized conformation of molecule (−)-cytisine squaramide **5**, in contrast to the crystal conformation, exhibits a twofold symmetry axis that runs through the centers of the C–C bonds of squaric acid (C14–C17 and C15–C16); both skeletons of (−)-cytisine groups exhibit identical geometrical parameters related by the twofold symmetry axis (Table 6).

**Figure 14:**
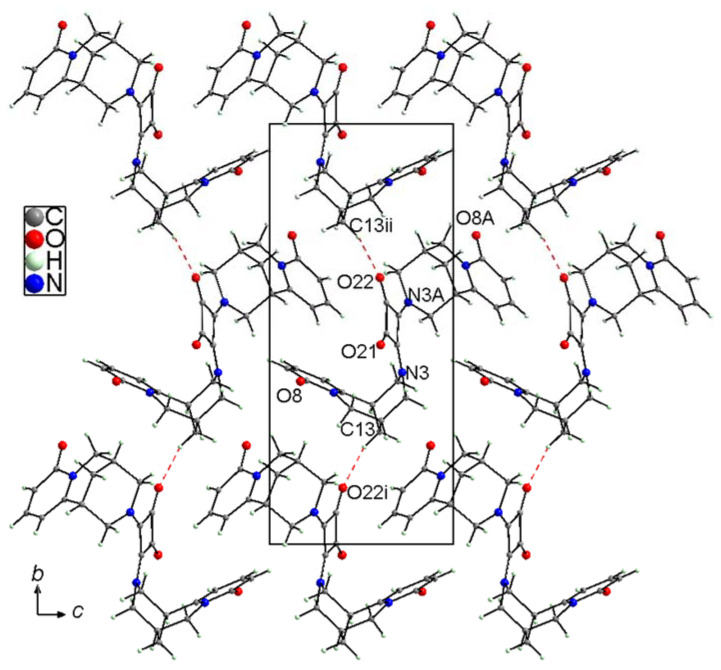
Packing diagram of **5** showing weak C–H⋯O contacts.

The arrangement of (−)-cytisine squaramide **5** molecules in the crystal is mainly determined by van der Waals forces and electrostatic interactions between molecular fragments characterized by the opposite EP signs. Additionally, the molecules related by a twofold screw axis are linked into chains along the *b*-axis through weak C–H⋯O interactions (Figure 14). The H⋯O distance is approximately 2.41(2) Å, and the C–H⋯O angle is about 140°. Between these chains, such interactions are significantly weaker over much longer distances.

### 2.3. Spectroscopic Analysis

**Figure 15:**
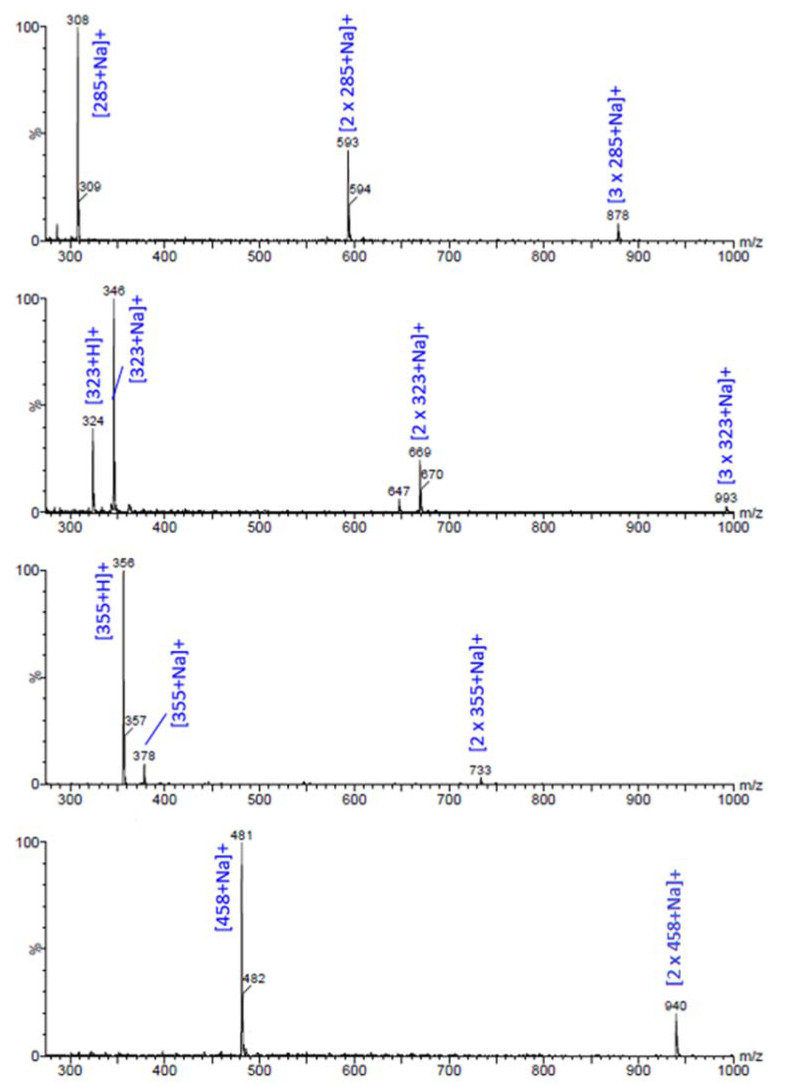
ESI-MS analyses of the new cytisine squaramides: **2** (MW 285), **3** (MW 323), **4** (MW 355) and **5** (MW 458).

The ESI MS spectra (Figure 15) show the presence of the desired squaramides (**2**–**5**), confirming that the new compounds have been successfully obtained. The mass-to-charge ratio (*m*/*z*) assigned to the signals implies the 1:1 stoichiometry of metal ion complexes [L + Me]^+^, which also demonstrates that the introduced compounds can easily coordinate alkali metal ions in a 1:1 stoichiometry with single ligands [L + Na]^+^ and in a 2:1 stoichiometry with dimers [2L + Na]^+^. Moreover, all derivatives (**2**–**5**) were observed to be more prone to dimerization.

Interestingly, however, among the four newly obtained cytisine squaramides (**2**–**5**), compounds **2** (primary amine) and **3** (secondary amine), with -NH_2_ and -N-propargyl substituents, respectively, differed from the other bis-squaramides. They easily formed not only the dimer [2L + Na]^+^ but also a trimer, which coordinates one Na^+^ ion [3L+Na]^+^. (**2** and **3**; Figure 15). It is also noteworthy that di-squaramide with morpholine substituent (**3**) shows low tendency to coordinate sodium ions, and ESI-MS spectrum shows an intense signal of the [L + H]^+^ ion.

**Figure 16:**
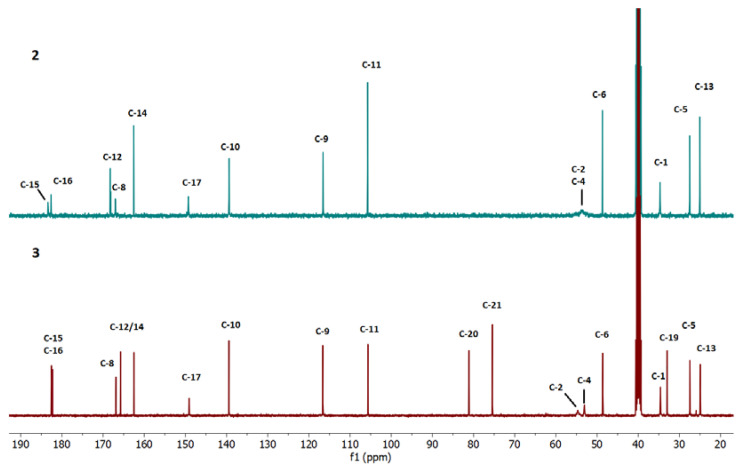
^13^C-NMR—comparison of the chemical shifts of compounds **2** and **3** in DMSO-d_6_.

**Figure 17:**
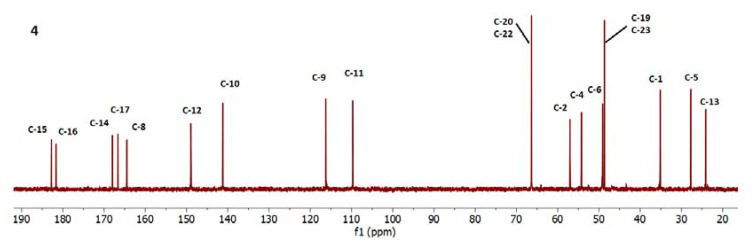
^13^C-NMR of compound **4** in D_2_O.

**Figure 18:**
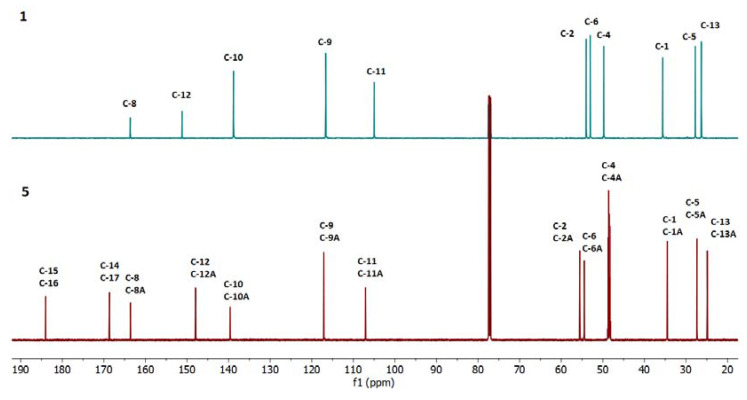
^13^C-NMR—comparison of the chemical shifts of (−)-cytisine (**1**) and (**5**) in CDCl_3_-MeOD (1:0.5 ratio).

In the next step of spectroscopic analyses, NMR spectra were recorded, and the results are presented below (Figure 16, Figure 17 and Figure 18 ), as well as in Table 7. Measurements were conducted in different solvents due to differences in the solubility of the respective squaramides. All data confirm the successful synthesis of novel compounds. The ^1^H-NMR spectra of compounds **2** and **3** (Supplementary Material) are significantly affected by hydrogen interactions involving protons at the N-18 nitrogen atoms, resulting in blurred and overlapping broad signals.

These superimposed signals pose challenges in assignment of chemical shifts in the ^1^H NMR analyses. The measured spectra of new compounds **2**, **3, 4**, and **5** are included in the Supplementary Section. Moreover, due to the hydrogen bonds of the amine groups, the ^13^C NMR spectra of compounds **2** and **3** exhibit faint and distorted signals of very low intensity (Figure 16, between 50–60 ppm). These signals correspond to the chemical shifts of the C-2 and C-4 carbon atoms in ring C of the cytisine molecule and may be influenced by proton interactions with N-18 nitrogen atom. In contrast, the ^1^H-NMR and ^13^C-NMR spectra of bisquaramide-cytisinium (**5**, Figure 18) reveal a perfectly symmetric molecule.

However, the ^1^H-NMR spectrum of squaramide **4** (Supplementary Material), featuring a morpholinium substituent, suggests the presence of this conjugate in conformational equilibrium in solution. This equilibrium results in superimposed signals in the proton spectrum, making it impossible to precisely assign chemical shifts to individual protons. Furthermore, the spectrum is undoubtedly influenced by hydrogen bonds (from H_2_O molecules), which may significantly affect the recorded signals, particularly the spectrum of compound **4** that was measured in D_2_O.

**Figure 19:**
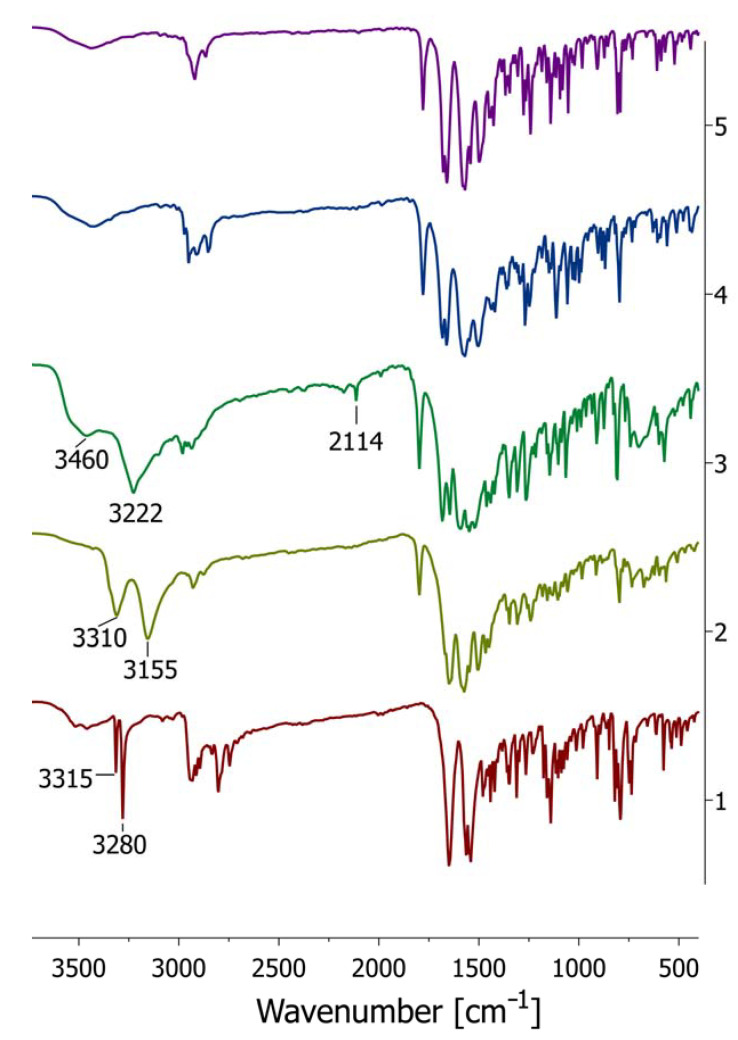
The overlapped FT–IR spectra of cytisine **1** and crystal of cytisine squaramides **2**, **3**, **4,** and **5** in KBr tablets in the range 3750–400 cm^−1^.

The FT–IR spectrum of cytisine **1** was compared to those of cytisine squaramides: **2**, **3**, **4**, **5** (Figure 19). The same overlapped spectra, presented on an extended scale for the characteristic vibrational ranges of ν(C=O, νC=C), are shown in Figure 19. In the FT-IR spectrum of the starting compound cytisine **1**, the bands assigned to the stretching vibration ν(N−H) appear relatively intense and narrow at 3315 cm^−1^, 3280 cm^−1^. Additionally, the stretching vibration ν(C=O) is observed at 1649 cm^−1^. These ν(N−H) bands completely disappear in the spectra of all cytisine squaramides due to the reaction occurring at the N3 atom of cytisine.

For squaramide **2**, which contains an NH_2_ group, two stretching vibrations of NH_2_ are observed: ν(N-H)_asym_ at 3310 cm^−1^ and ν(N-H)_sym_ at 3155 cm^−1^. In the IR spectrum of squaramide **3**, the ν(≡C-H) and ν(C≡C) stretching modes of propargyl moiety have been assigned to the intense band at 3222 cm^−1^ and low intense band at 2114 cm^−1^, respectively. The ν(≡C-H) band overlaps the ν(N-H) vibration band. Additionally, the broad band near 3460 cm^−1^ is attributed to the ν(O-H) vibrations from water molecules present in the crystal structure of compound **3**.

**Figure 20:**
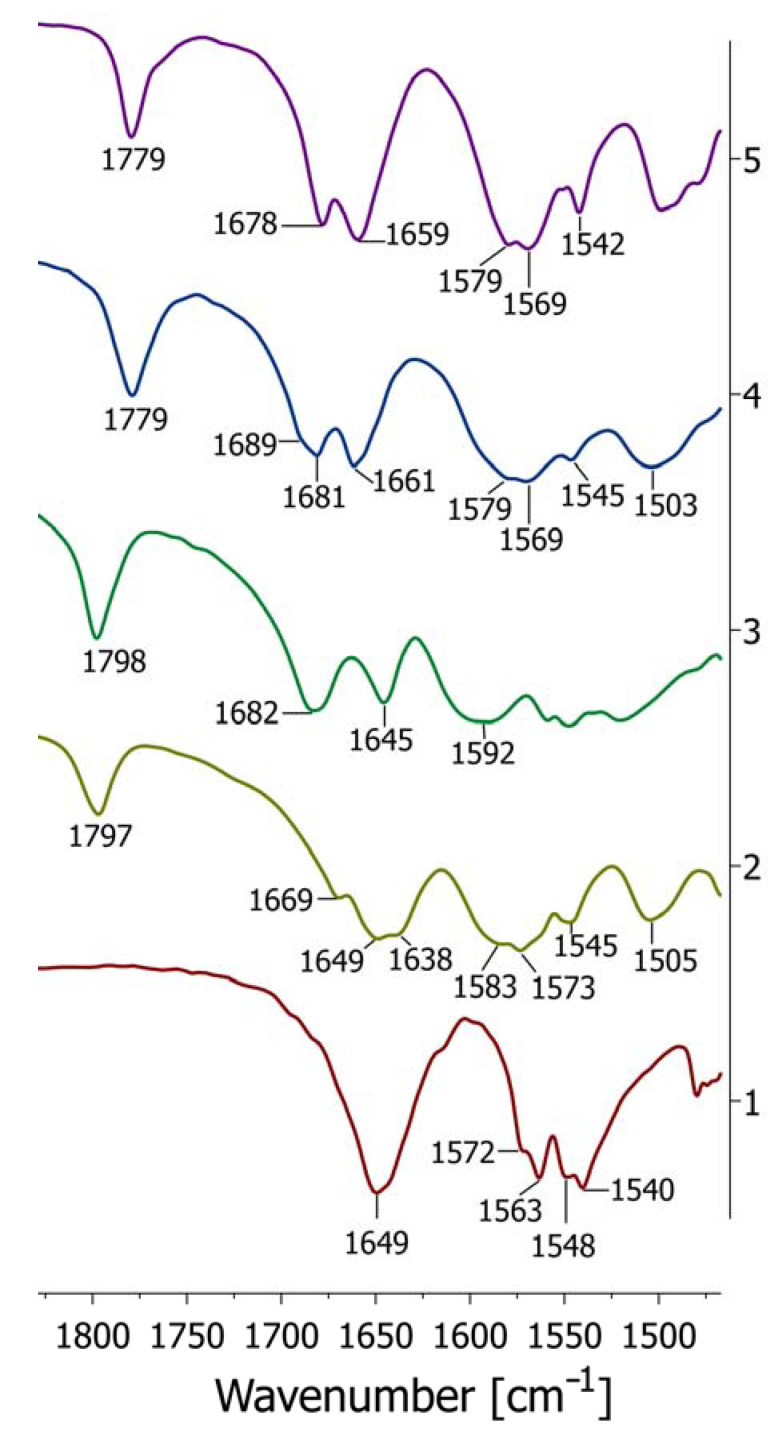
The overlapped FT–IR spectra of cytisine **1** and crystal of cytisine squaramides **2**, **3**, **4,** and **5** in KBr tablets in the range 1825–1450 cm^−1^.

The analysis of the IR spectra (Figure 20) of cytisine squaramides **2**, **3**, **4**, and **5** revealed two characteristic intense bands associated with the squaramide structure. These bands appear in the ranges 1779–1798 cm^−1^ and 1669–1689 cm^−1^, corresponding to the asymmetric and symmetric stretching vibrations of the C=O moiety, respectively. The ν(C=O) stretching vibration is coupled with ν(C=C) stretching mode. However, the band assigned to ν(C=C) vibrations is overlapped by that assigned to the ν(C=O) of the cytisine moiety.

## 3. Materials and Methods

### 3.1. General

*Spectroscopic measurements: The ESI mass spectra* were obtained on a Waters/Micromass (Manchester, UK) ZQ mass spectrometer (MassLynx V4.0). The samples were prepared in methanol or in methanol–water solution. The concentration of the analyzed samples was 2 × 10^−5^ mol/dm^3^ (1:1 ratio), which is typical of ESI, unless indicated otherwise.

The FT-IR spectra of each compound were recorded in KBr tablets on an IFS 113 v FT-IR spectrophotometer (Bruker, Karlsruhe, Germany) equipped with a DTGS detector; wavenumbers (cm^–1^) resolution = 2 cm^–1^, NSS = 125, description w = weak, m = medium, s = strong, br = broad. The Happ–Genzel apodization function was used.

*The NMR spectra* of the product were recorded using Bruker Ascend 600 MHz spectrometer. The ^1^H-NMR measurements were carried out at an operating frequency of 600.14 MHz (pulse sequence = zg30, number of scans = 32), while the ^13^C NMR spectra were recorded at an operating frequency of 150.91 MHz (pulse sequence = zg30, number of scans = 1024). No window function or zero filling was used. The errors of ^1^H and ^13^C-NMR chemical shift values were 0.01 ppm and 0.1 ppm, respectively.

Optical rotation was measured using a Perkin Elmer (Waltham, MA, USA) 243B polarimeter at 20 °C in ethanol solution, with a 100 mm cell, Na lamp D-line under 20 °C.

### 3.2. Synthesis of New (−)-Cytisine Squaramides (***2*-*5***)

(−)-Cytisine (**1**, 1.9 g, 10 mmoles; [α]^2^⁰/D = −110° at c = 1 in ethanol) was dissolved in absolute ethanol, and diethylsquarate (**B**, 1.7 g, 10 mmoles, [24]) was added at a molar ratio 1:1, yielding the monoamide cytisine-ethyl squarate. The reaction proceeded overnight at room temperature, achieving an 80% yield (2.51 g).

Initially, to accelerate the process, experiments were conducted at elevated temperatures under reflux. However, TLC analysis and then ESI-MS spectra revealed a mixture of compounds, including compound **5**, squaramide acid, squaric acid, and several unidentified by-products. Given these results, the reaction was instead carried out for a longer duration at room temperature, which proved to be more favorable for the reaction’s progress and improved the overall yield.

In the next step, monoamide cytisine-ethyl squarate was dissolved in 96% ethanol, and the appropriate amine: NH_3_(aq), propargyl amine, and morpholine was added in a 1:1 molar ratio. Each amine was introduced in a separate reaction. The reaction proceeded overnight at room temperature, yielding products **2**, **3**, and **4**, respectively (Scheme 2).

The reaction of (−)-cytisine with diethyl-squarate in abs. ethanol at a 2:1 molar ratio proceeded overnight at room temperature, resulting in the formation of bis-cytisine squaramide (**5**, Scheme 2).

The reaction mixtures were purified using flash-chromatography on silica gel (SiO_2_) columns, employing a mobile phase of CH_2_Cl_2_:MeOH:NH_4_OH in a 90:10:1 ratio. The structures of the new squaramides were confirmed by ESI-MS, which showed molecular ion peaks consistent with the expected products (Figure 15), as well as by ^1^H and ^13^C NMR spectra (Figure 16, Figure 17 and Figure 18, Table 7) and FT-IR (Figure 19 and Figure 20). The obtained compounds were recrystallized from the mixture ethanol/2-propanol and analyzed using single-crystal X-ray diffraction techniques. Melting point analyses indicated that all the synthesized conjugates **2**, **3**, **4**, and **5** decompose upon increasing temperature.

**Squaramide 2:** starting from monoamide cytisine-ethyl squarate (1 mmol, 0.314 g) and 4 mL of NH_4_OH (25%), yielding 0.128 g (45%) of new compound; C_15_H_15_N_3_O_3_, MW 285.30; ESI-MS: [285 + Na]^+^; ^1^H-NMR (400 MHz, DMSO-d_6_) δ (ppm): δ 7.55 (1H, broad signal of N-H), 7.33 (1H, dd), 6.19 (1H, dd), 6.13 (1H, dd), 4.55 (1H, broad signal of N-H), 4.00 (1H, m), 3.69 (1H, dd), 3.52–3.40 (4H, broad overlap signal, m), 3.18 (1H, s), 2.46 (1H, m), 2.01 (2H, m), and ^13^C-NMR (150 MHz, DMSO-d_6_, Figure 16, Table 7), ${\left[\alpha \right]}_{D}^{20}=$ –38° (c = 1 in ethanol).

**Squaramide 3**: starting from monoamide cytisine-ethyl squarate (1 mmol, 0.314 g), yielding 0.184 g (57%) of new compound; C_18_H_16_N_3_O_3_, MW 323.35; ESI-MS: [323 + H]^+^, [323 + Na]^+^; ^1^H-NMR (400 MHz, DMSO-d_6_) δ (ppm): δ 7.90 (1H, broad s, N-H), 7.32 (1H, dd), 6.20 (1H, d), 6.13 (1H, d), 4.38–4.23 (2H, broad 2 overlap signals, m), 3.97 (1H, d), 3.69 (1H, ddd), 3.52 (1H, ddd), 3.44 (1H, ddd), 3.30 (1H, t), 3.19 (1H, s), 2.51 (2H, m), 2.01 (2H, m), and ^13^C-NMR (150 MHz, DMSO-d_6_, Figure 16, Table 7), ${\left[\alpha \right]}_{D}^{20}=$ –169° (c = 1 in ethanol).

**Squaramide 4**: starting from monoamide cytisine-ethyl squarate (1 mmol, 0.314 g), yielding 0.188 g (53%) of new compound; C_19_H_21_N_3_O_4_, MW 355.39; ESI-MS: [355 + H]^+^ [355 + Na]^+^; ^1^H-NMR (400 MHz, D_2_O) δ (ppm): 7.49 (1H, dd), 6.47 (1H, d), 6.40 (1H, d), 4.20 (2H, t), 4.17, 3.85–3.57 (broad area of overlap signals; 8H), 3.51 (3H, m), 3.33 (1H, m), 3.16 (1H, m), 2.57 (1H, m), 2.10 (2H, m) and^13^C-NMR (150 MHz, D_2_O, Figure 17, Table 7), ${\left[\alpha \right]}_{D}^{20}=$ –384° (+/−5) (c = 0.5 in ethanol).

**Squaramide 5:** starting from cytisine (0.38 g, 2 mmoles) and ethyl squarate (1 mmol; 0.170 g), yielding 0.298 g (65%) of new compound; C_26_H_26_N_4_O_4_, MW 458.52; ESI-MS: [458 + Na]^+^; ^1^H-NMR (400 MHz, CDCl_3_-MeOH 1:0.5) δ (ppm): 7.32 (2H, dt), 6.45 (2H, dd), 6.17 (2H, t), 4.28 (2H, d), 4.25 (2H, dd), 3.96 (4H, dt), 3.82 (2H, dt), 3.55 (2H, dq), 3.46 (2H, m), 3.35 (MeOD), 3.21 (2H, m), 2.57 (2H, m), 2.11 (2H, m), and ^13^C-NMR (150 MHz, CDCl_3_-MeOH, Figure 18, Table 7), ${\left[\alpha \right]}_{D}^{20}=$ –442° (+/−2) (c = 1 in ethanol).

### 3.3. X-Ray Single Crystal Data Collection

The X-ray intensity data for selected single crystals of the investigated compounds were collected using graphite monochromatic Mo Kα radiation on a four-circle κ geometry Xcalibur diffractometer with a Sapphire2 area CCD detector. Data were collected using the CrysAlisPro 1.171.42.93a. Integration, scaling of the reflections, corrections for Lorenz, and polarization effects and absorption corrections were performed using the CrysAlisPro 1.171.42.93a program [25]. Using Olex2-1.51 [26], the structures were solved by the direct methods using SHELXT-2014/7 [27] and refined using the SHELXL-2018/3 program [28].

The hydrogen atoms joined to carbon atoms were introduced in their geometrical positions and treated as rigid. The H atoms involved in the hydrogen bonds were refined, provided that they gave reasonable hydrogen bonds; otherwise, they were constrained. The final difference Fourier maps showed no peaks of chemical significance. Collection data and structure refinement, as well as details of the crystal, are presented in Table 8. The structures were visualized with Diamond 3.0 [29]. Deposition Numbers: 2421174, 2421175, 2421176, 2421177 contain the supplementary crystallographic data for this paper for compounds **2**, **3**, **4,** and **5**, respectively. These data can be obtained free of charge via the joint Cambridge Crystallographic Data Centre (CCDC) and Fachinformationszentrum Karlsruhe Access Structures (FIZ) service.

### 3.4. DFT Calculation

To gain a comprehensive understanding of the electronic structure and properties of the investigated compounds, a series of density functional theory (DFT) calculations were performed. Geometries of the investigated compounds were optimized using the Gaussian09 software package [30]. The optimization process was conducted without any symmetry constraints to achieve the most stable structure. The Becke, three-parameter, Lee-Yang-Parr (B3LYP) exchange-correlation functional was employed, along with the 6-311++G(*d*,*p*) basis set for all atoms [31,32]. This level of theory has been chosen for its balance between computational efficiency and accuracy in predicting molecular geometries. The optimized molecular structure was visualized using GaussView program (*Gaussian09)* [33], enabling a detailed examination of bond lengths, angles, and overall molecular conformation. Additionally, the three-dimensional molecular electrostatic potential maps were generated to elucidate intermolecular interactions.

## 4. Conclusions

Squaric acid, thoroughly investigated from the viewpoints of chemistry, pharmaceutics, and materials science, is known for its high acidity due to resonance stabilization. Its derivatives, especially squaramides, have diverse applications, including antiparasitic, antibacterial, cytotoxic, and antiviral activities. They are also explored for drug discovery, cancer therapies, and environmental applications like water purification and toxin detection.

A synthesis method of cytisine squaramides under efficient, low-cost, and mild conditions has been developed. Novel cytisine-squaramide conjugates (**2**–**5**) were synthesized using diethyl squarate, cytisine, and amines such as ammonia, propargylamine, and morpholine. Comprehensive structural and spectroscopic analyses, including ESI-MS, NMR, FT-IR, X-ray crystallography, and DFT calculations, confirmed successful synthesis and provided detailed insights into molecular conformations, hydrogen bonding, and electrostatic interactions.

The combined single-crystal analyses and DFT studies revealed the structural diversity of cytisine squaramide derivatives, emphasizing the influence of substituents and intermolecular interactions. These findings provide critical insights into their stability and behavior in both solid and solution states, offering potential applications in drug design and material sciences.

The squaramide derivatives studied exhibit diverse crystal structures and intermolecular interactions, primarily governed by hydrogen bonding, electrostatic forces, and π-stacking. For example, compound **2** crystallizes in a triclinic system with two distinct conformers (A and B) per unit cell. Its structure is dominated by hydrogen bonding, with dimers formed via N–H⋯O interactions stabilized by an R₂^2^(10) motif, extending into chains through translationally related hydrogen bonds. Despite a 180° rotation of the squaramide group in molecule B relative to its position in molecule A, DFT optimization indicates that molecule A is slightly more stable. The dimer stabilization energy (~67.75 kJ/mol) highlights the significance of hydrogen bonding in maintaining structural integrity.

In contrast, squaramide **3**·H₂O introduces water-mediated hydrogen bonding as a defining feature. Crystallizing as a monohydrate in the orthorhombic system, water molecules act as both donors and acceptors, forming hydrogen-bonded ribbons along the a-axis. Steric effects from the bulky N-propargyl substituent induce conformational changes in the piperidine ring, influencing crystal packing. DFT calculations closely align with experimental results, emphasizing the role of molecular electrostatic potential in crystal growth and stabilization.

Squaramide **4**, crystallizing in a monoclinic system, features two independent molecules per an asymmetric unit. Weak C–H⋯O contacts and van der Waals forces primarily stabilize the structure. Differences in the tilt of the pyridine and squaramide rings, reflected in dihedral angles of 15.6° and 26.4°, are not observed in the DFT-optimized structure, where both molecules adopt identical conformations. This suggests that packing forces contribute to the observed experimental deviations.

Similarly, squaramide **5**, also crystallizing in a monoclinic system, contains a single molecule per asymmetric unit. Although the DFT-optimized structure suggests a twofold symmetry, the experimental crystal structure shows deviations likely caused by packing interactions within the lattice. The stability of the structure is maintained by weak C–H⋯O hydrogen bonds and van der Waals forces, which form chains along the b-axis. These findings highlight the crucial roles of dispersion and electrostatic interactions in maintaining the overall structural cohesion.

Overall, the study reveals that the stability of cytisine-squaramides **2**–**5** is predominantly influenced by hydrogen bonding, complemented by electrostatic and van der Waals interactions. Structural variations, such as water incorporation or steric hindrance from substituents, significantly affect conformational preferences and packing arrangements. The strong agreement between the experimental and DFT-optimized geometries demonstrates the reliability of computational methods in predicting and elucidating these structural properties.

## Figures and Tables

**Table 1:** Hydrogen-bond geometry (Å, °) in crystal **2**.

***D***—H⋯***A***	***D***—H	H⋯***A***	***D***⋯***A***	***D***—H⋯***A***
N18A—H18A⋯O16B	0.88 (4)	2.03 (4)	2.870 (5)	160 (4)
N18A—H18B⋯O15A *^i^*	0.93 (6)	2.03 (6)	2.955 (5)	178 (5)
N18B—H18C⋯O16A	0.96 (4)	1.94 (5)	2.890 (5)	169 (4)
N18B—H18D⋯O15B *^ii^*	0.88 (5)	2.09 (5)	2.935 (5)	161 (4)

Symmetry codes: ^(^*i*^)^ *x* + 1, *y*, *z*; ^(*ii*)^ *x −* 1, *y*, *z.*

**Table 2:** Selected conformational parameters for (−)-cytisine squaramide **2**.

	X-Ray		DFT	
	Mol A	Mol B	Mol A	Mol B
C1—C2—N3—C4	55.9 (5)	56.1 (5)	53.69	54.19
C1—C2—N3—C14	−113.4 (4)	−121.3 (4)	−140.66	−129.52
C2—N3—C14—C15	−15.9 (7)	−179.7 (4)	1.79	−166.54
C2—C1—C12—C11	86.3 (5)	84.6 (5)	87.46	88.41

**Table 3:** Selected conformational parameters for (−)-cytisine squaramide **3**.

	X-Ray	DFT
C1—C2—N3—C4	−56.9 (3)	−51.80
C1—C2—N3—C14	102.8 (3)	144.73
C2—N3—C14—C15	9.1 (4)	−13.59
C2—C1—C12—C11	−82.4 (3)	−87.29

**Table 4:** Hydrogen bond geometry (Å, º) in crystal **3‧**H_2_O.

***D***—H⋯***A***	***D***—H	H⋯***A***	***D***⋯***A***	***D***—H⋯***A***
O1*W*—H1WB⋯O8	0.85	1.93	2.775 (3)	170
N18—H18⋯O1W *^i^*	0.86	2.11	2.935 (3)	160

Symmetry codes: ^(*i*)^ *x* + 1/2, −*y* + 1/2, −*z* + 1.

**Table 5:** Selected conformational parameters for (−)-cytisine squaramide **4**.

	X-Ray		DFT
	Mol A	Mol B	
C1—C2—N3—C4	54.1 (5)	53.8 (5)	49.73
C1—C2—N3—C14	−101.1 (4)	−108.6 (5)	−128.14
C2—N3—C14—C15	143.3 (5)	150.9 (5)	158.30
C2—C1—C12—C11	78.0 (5)	86.1 (5)	88.49

**Table 6:** Selected conformational parameters for (−)-cytisine squaramide **5**.

	X-Ray	DFT
C1—C2—N3—C4	51.2 (3)	49.50
C1A—C2A—N3A—C4A	51.5 (3)	49.50
C1—C2—N3—C14	−114.8 (2)	−134.76
C1A—C2A—N3A—C17	−125.1 (2)	−134.76
C2—N3—C14—C15	146.3 (3)	161.00
C2A—N3A—C17—C16	153.7 (3)	161.00
C2—C1—C12—C11	93.1 (3)	88.41
C2A—C1A—C12A—C11A	87.2 (3)	88.41

**Table 7:** Summary of the chemical shifts of carbon atoms in the measured ^13^C NMR (δ [ppm]) of cytisine (**1**) and its squaramides: **2** and **3** in DMSO-d_6_, **4** in D_2_O, and **5** in CDCl_3_.

C Atoms	2 DMSO	3 DMSO	4 D_2_O	5 CDCl_3_/MeOD (1:0.5)	1 CDCl_3_
1	27.5	27.5	35.1	33.5 (1 and 1A)	35.4
2	53.9 ^a^	54.7 ^a^ #	57.0	55.2 (2 and 2A)	53.8
4	53.9 ^a^	53.1 ^a^ #	49.1	48.7 (4 and 4A)	49.5
5	34.7	34.6	27.7	27.4 (5 and 5A)	27.5
6	48.7	48.7	54.2	54.5 (6 and 6A)	52.8
8	167.0	166.9	164.5	163.6 (8 and 8A)	163.4
9	116.6	116.7	116.2	117.1 (9 and 9A)	116.4
10	139.4	139.5	141.2	139.6 (10 and 10A)	138.6
11	105.8	105.7	109.7	107.1 (11 and 11A)	104.7
12	168.3	165.8	149.0	147.9 (12 and 12A)	150.9
13	25.1	25.0	24.1	24.9 (13 and 13A)	26.1
14	162.6	162.5	168.0 (or 17)	168.7 (14 and 17)	
15	183.4	182.6 *	182.8 (or 16)	184.0 (15 and 16)	
16	182.7	182.5 *	181.6 (or 15)	184.0 (16 and 15)	
17	149.3	149.1	166.6 (or 14)	168.7 (17 and 14)	
19		33.0	48.7 (19/23)		
20		81.1	66.3 (20/22)		
21		75.5	-		

^a^—two overlapped signals with very low intensities; * or #—interchangeable signals.

**Table 8:** Crystal data and details of data collection and final refinement parameters.

	2	3	4	5
CCDC No.	2421174	2421175	2421176	2421177
Formula	C_15_H_15_N_3_O_3_	C_18_H_17_N_3_O_3_•H_2_O	C_19_H_21_N_3_O_4_	C_26_H_26_N_4_O_4_
Molecular weight	285.30	341.36	355.39	458.51
Temperature (K)	295 (2)	295 (2)	295 (2)	100 (2)
Crystal system	triclinic	orthorhombic	monoclinic	monoclinic
Space group	P1	P 2_1_2_1_2_1_	P 2_1_	P 2_1_
a (Å)	6.9269 (5)	8.2405 (3)	10.7027 (5)	6.9626 (2)
b (Å)	7.7099 (6)	12.6825 (5)	7.4702 (3)	18.6664 (6)
c (Å)	13.9934 (9)	16.3143 (7)	22.0755 (11)	8.6303 (3)
α (^o^)	92.495 (5)			
β (^o^)	103.055 (6)		102.381 (4)	109.095 (4)
γ (^o^)	110.026 (5)			
V (Å^3^)	678.00 (9)	1705.01 (12)	1723.92 (14)	1059.94 (6)
Z	2	4	4	2
F(000)	300	720	752	484
D_cal_ (g^.^cm^−1^)	1.397	1.330	1.369	1.437
θ range(^o^)	2.837–29.288	2.769–29.312	2.886–29.448	2.497–29.380
μ (mm^−1^)	0.100	0.096	0.098	0.099
Crystal size (mm)	0.26 × 0.21 × 0.13	0.28 × 0.26 × 0.15	0.29 × 0.26 × 0.19	0.29 × 0.25 × 0.14
T_min_/T_max_	0.965/1.00	0.980/1.00	0.974/1.00	0.977/1.00
Total/unique/obs. refls (F^2^ > 2σ(F^2^))	10596/6186/3886	12022/4143/2642	28225/8269/5037	23797/5253/4470
R_int_	0.0273	0.0331	0.0493	0.0473
R [F^2^ > 2σ(F^2^)] ^(*)^	0.0543	0.0481	0.0673	0.0422
wR [F^2^ all refls] ^(*)^	0.1114	0.1067	0.1475	0.0899
S	1.020	1.033	1.002	1.002
Flack parameter	−1.7 (10)	−0.2(3)	−0.5(7)	−0.1(3)
Δρ_max_, Δρ_min_ (eÅ^−3^)	+0.196, −0.172	+0.157, −0.138	+0.216, −0.180	+0.264, −0.227

^(*)^ *R* = Σ ||*F*_o_|–|*F*_c_||/Σ*F*_o_, *wR* = {Σ [*w*(*F*_o_^2^–*F*_c_^2^)^2^]/Σ*wF*_o_^4^}^½^; *w*^–1^ = σ^2^(*F*_o_^2^) + (*aP*)^2^ + *bP* where *P* = (*F*_o_^2^ + 2*F*_c_^2^)/3. The a and b parameters are 0.0392 and 0.0169 for 2, 0.0436 and 0.0185 for 3, 0.0520 and 0.6949 for 4, 0.406 and 0.2174 for 5.

## Data Availability

Dataset available on request from the authors.

## Supplementary Materials

The following supporting information can be downloaded at: https://www.mdpi.com/article/10.3390/molecules30051135/s1, Table S1. (a) Optimized parameters for (−)-cytisine squaramide 2–Mol_A, (b) Optimized parameters for (−)-cytisine squaramide **2**–Mol_B, (c) Optimized parameters for (−)-cytisine squaramide **2** dimeric structure (AB); Table S2. Optimized parameters for (−)-cytisine squaramide **3**; Table S3. Optimized parameters for (−)-cytisine squaramide **4**; Table S4. Optimized parameters for (−)-cytisine squaramide **5**; Figure S1. ^1^H NMR and ^13^C NMR spectra of (−)-cytisine squaramide **2** in DMSO-d_6_; Figure S2. ^1^H NMR and ^13^C NMR spectra of (−)-cytisine squaramide **3** in DMSO-d_6_; Figure S3. ^1^H NMR and ^13^C NMR spectra of (−)-cytisine squaramide **4** in D2O; Figure S4. ^1^H NMR and ^13^CNMR spectra of (−)-cytisine squaramide **5** in CDCl_3_:MeOD (1:0.5).
