# Supplementary material for: Synthesis and Structural Analysis of New (−)-Cytisine Squaramides
Source: Molecules. 2025 Mar 1;30(5):1135. doi: 10.3390/molecules30051135 (PMC11901779; doi:10.3390/molecules30051135)
Supplement: Supplementary file 1 [file molecules-30-01135-s001.zip › molecules-3486266-supplementary.pdf]

Supplementary Materials

Synthesis and structural analysis of new (−)-cytisine squaramides

Anna K. Przybył[a,*], Jan Janczak[b], Adam Huczyński[a,*]

[a] *Department of Medical Chemistry, Faculty of Chemistry, Adam Mickiewicz University, Uniwersytetu Poznańskiego 8, 61-614, Poznań, Poland;*

[b] *Institute of Low Temperature and Structure Research, Polish Academy of Sciences, Okólna 2 str., 50–422, Wrocław, Poland*

Content

Table S1. (a) Optimized parameters for (−)-cytisine squaramide **2** – Mol_A.

Table S1. (b) Optimized parameters for (−)-cytisine squaramide **2** – Mol_B.

Table S1. (c) Optimized parameters for (−)-cytisine squaramide **2** dimeric structure (AB).

Table S2. Optimized parameters for (−)-cytisine squaramide **3**.

Table S3. Optimized parameters for (−)-cytisine squaramide **4**.

Table S4. Optimized parameters for (−)-cytisine squaramide **5**.

Figure S1. ^{1}H-NMR (600 MHz) and ^{13}C-NMR (150 MHz) spectra of (−)-cytisine squaramide **2** in DMSO-d_6.

Figure S2. ^{1}H-NMR (600 MHz) and ^{13}C-NMR (150 MHz) spectra of (−)-cytisine squaramide **3** in DMSO-d_6.

Figure S3. ^{1}H-NMR (600 MHz) and ^{13}C-NMR (150 MHz) spectra of (−)-cytisine squaramide **4** in D_2O.

Figure S4. ^{1}H-NMR (600 MHz) and ^{13}C-NMR (150 MHz) spectra of (−)-cytisine squaramide **5** in $CDCl_3$:CD_3OD (1:0.5).

Table S1. (a) Optimized parameters for (−)-cytisine squaramide **2** – Mol_A.

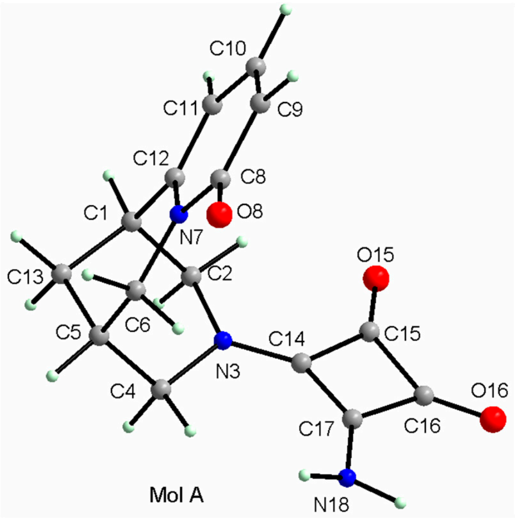

Bonds (Å)					
C1–C2	1.546	C2–N3	1.466	N3–C4	1.463
C4–C5	1.539	C5–C6	1.530	C5–C13	1.533
C13–C1	1.533	C6–N7	1.480	N7–C8	1.427
C8–C9	1.440	C9–C10	1.363	C10–C11	1.416
C11–C12	1.369	C12–C1	1.516	C12–N7	1.375
C8–O8	1.229	N3–C14	1.347	C14–C15	1.499
C15–O15	1.208	C15–C16	1.535	C16–O16	1.209
C16–C17	1.476	C17–C14	1.398	C17–N18	1.355
C1–H	1.093	C4–H	1.095	C5–H	1.095
C6–H	1.093	C13–H	1.094	Car–H	1.083
N18–H	1.009				
**Angles (°)**					
C1–C2–N3	110.14	C2–N3–C4	114.91	N3–C4–C5	111.61
C4–C5–C6	113.26	C4–C5–C13	110.10	C5–C13–C1	106.70
C13–C1–C2	109.21	C5–C6–N7	114.80	C6–N7–C8	113.27
N7–C8–O8	119.13	N7–C8–C9	115.09	C8–C9–C10	121.41
C9–C10–C11	120.70	C10–C11–C12	119.82	C11–C12–C1	121.22
C11–C12–N7	119.90	C12–N7–C8	123.07	C2–N3–C14	120.12
C4–N3–C14	123.26	N3–C14–C15	131.33	C14–C15–O15	134.79
C14–C15–C16	87.19	C15–C16–O16	138.65	C15–C16–C17	87.52
C16–C17–N18	128.29	N18–C17–C14	138.29	C16–C17–C14	93.38
**Torsion angles (°)**					
C1–C2–N3–C4	53.69	C2–N3–C4–C5	-51.93	N3–C4–C5–C6	-67.80
N3–C4–C5–C13	55.10	C4–C5–C13–C1	-60.61	C5–C13–C1–C2	-59.86
C4–C5–C6–N7	82.71	C5–C6–N7–C8	-172.74	C6–N7–C8–O8	2.37
C6–N7–C8–C9	-177.41	N7–C8–C9–C10	1.73	C8–C9–C10–C11	-0.76
C9–C10–C11–C12	-0.67	C10–C11–C12–C1	-176.71	C10–C11–C12–N7	0.80
C11–C12–C1–C2	87.47	C12–N7–C8–O8	178.26	C1–C2–N3–C14	-140.66
C2–N3–C14–C15	1.79	N3–C14–C15–O15	1.45	N3–C14–C15–C16	-179.65
C14–C15–C16–O16	178.28	C14–C15–C16–C17	-1.53	O15–C15–C16–O16	-2.88
C15–C16–C17–N18	179.61	C15–C16–C17–C14	1.62	O16–C16–C17–N18	-0.22
O16–C16–C17–C14	178.15	N18–C17–C14–C15	-179.28	C17–C14–C15–O15	-177.29
N18–C17–C14–N3	2.10	C17–C14–N3–C4	-15.64		

Direct from Gaussian

Mol A

Center Number	Atomic Number	Atomic Type	Coordinates (Angstroms) X	Y	Z
1	6	C	-0.095897	-0.080970	0.033576
2	1	H	-0.635221	0.826285	-0.248578
3	6	C	-0.269189	-0.264329	1.558968
4	1	H	0.129795	0.626610	2.066017
5	1	H	-1.316535	-0.367359	1.834846
6	7	N	0.455075	-1.457061	2.009598
7	6	C	1.867998	-1.499310	1.632380
8	1	H	2.289382	-2.462313	1.920344
9	1	H	2.418439	-0.726288	2.189372
10	6	C	2.059313	-1.263024	0.123236
11	1	H	3.138124	-1.216614	-0.056118
12	6	C	1.513591	-2.402268	-0.740741
13	1	H	2.017009	-2.407676	-1.712039
14	1	H	1.716418	-3.379930	-0.299790
15	7	N	0.059285	-2.338485	-1.005335
16	6	C	-0.443926	-3.496122	-1.671172
17	8	O	0.322313	-4.425239	-1.917465
18	6	C	-1.845508	-3.450371	-2.000488
19	1	H	-2.247164	-4.326628	-2.491745
20	6	C	-2.605677	-2.354976	-1.719432
21	1	H	-3.656824	-2.341114	-1.987323
22	6	C	-2.039147	-1.227507	-1.076292
23	1	H	-2.641270	-0.359605	-0.842868
24	6	C	-0.716888	-1.239259	-0.721396
25	6	C	1.396070	0.055054	-0.291880
26	1	H	1.548836	0.244082	-1.358395
27	1	H	1.825497	0.899793	0.255582
28	6	C	-0.084336	-2.262266	2.944774
29	6	C	-1.400101	-2.205298	3.660323
30	8	O	-2.360548	-1.474168	3.618369
31	6	C	-0.964463	-3.474386	4.404998
32	8	O	-1.387329	-4.213210	5.262874
33	6	C	0.276199	-3.440019	3.606219
34	7	N	1.325658	-4.295398	3.648596
35	1	H	1.181194	-5.133926	4.194980
36	1	H	1.938765	-4.404319	2.856817

Table S1. (b) Optimized parameters for (−)-cytisine squaramide **2** – Mol_B.

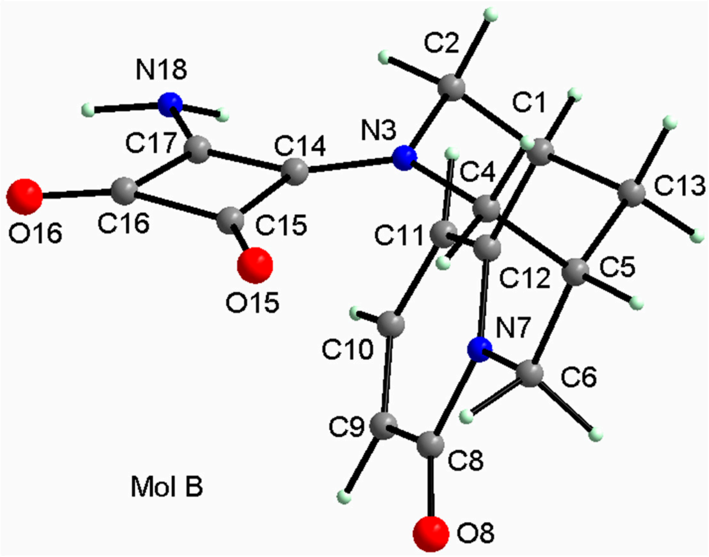

Bonds (Å)					
C1–C2	1.548	C2–N3	1.458	N3–C4	1.467
C4–C5	1.540	C5–C6	1.533	C5–C13	1.534
C13–C1	1.534	C6–N7	1.485	N7–C8	1.431
C8–C9	1.443	C9–C10	1.362	C10–C11	1.418
C11–C12	1.372	C12–C1	1.514	C12–N7	1.371
C8–O8	1.225	N3–C14	1.345	C14–C15	1.498
C15–O15	1.207	C15–C16	1.538	C16–O16	1.208
C16–C17	1.476	C17–C14	1.397	C17–N18	1.358
C1–H	1.093	C4–H	1.095	C5–H	1.094
C6–H	1.093	C13–H	1.094	Car–H	1.083
N18–H	1.011				
**Angles (°)**					
C1–C2–N3	110.77	C2–N3–C4	114.83	N3–C4–C5	110.49
C4–C5–C6	113.33	C4–C5–C13	109.99	C5–C13–C1	106.88
C13–C1–C2	109.15	C5–C6–N7	114.96	C6–N7–C8	113.25
N7–C8–O8	119.26	N7–C8–C9	115.10	C8–C9–C10	121.51
C9–C10–C11	120.56	C10–C11–C12	119.76	C11–C12–C1	121.03
C11–C12–N7	120.19	C12–N7–C8	122.82	C2–N3–C14	123.63
C4–N3–C14	121.43	N3–C14–C15	131.61	C14–C15–O15	134.65
C14–C15–C16	87.11	C15–C16–O16	138.53	C15–C16–C17	87.42
C16–C17–N18	129.38	N18–C17–C14	137.16	C16–C17–C14	93.45
**Torsion angles (°)**					
C1–C2–N3–C4	54.19	C2–N3–C4–C5	-53.58	N3–C4–C5–C6	-66.09
N3–C4–C5–C13	56.88	C4–C5–C13–C1	-61.40	C5–C13–C1–C2	-61.34
C4–C5–C6–N7	84.83	C5–C6–N7–C8	-175.52	C6–N7–C8–O8	1.20
C6–N7–C8–C9	-178.56	N7–C8–C9–C10	2.59	C8–C9–C10–C11	-0.98
C9–C10–C11–C12	-0.50	C10–C11–C12–C1	-177.59	C10–C11–C12–N7	0.18
C11–C12–C1–C2	88.41	C12–N7–C8–O8	176.81	C1–C2–N3–C14	-129.51
C2–N3–C14–C15	-166.54	N3–C14–C15–O15	-3.42	N3–C14–C15–C16	178.33
C14–C15–C16–O16	-177.76	C14–C15–C16–C17	2.01	O15–C15–C16–O16	4.11
C15–C16–C17–N18	179.25	C15–C16–C17–C14	-2.16	O16–C16–C17–N18	-0.96
O16–C16–C17–C14	177.63	N18–C17–C14–C15	-179.39	C17–C14–C15–O15	176.12
N18–C17–C14–N3	0.11	C17–C14–N3–C4	-169.82		

Direct from Gaussian

Mol B

Center Number	Atomic Number	Atomic Type	Coordinates (Angstroms) X	Y	Z
1	6	C	0.005822	0.002139	-0.015159
2	1	H	-1.049010	0.055374	0.265602
3	6	C	0.101927	0.271663	-1.536843
4	1	H	-0.510234	-0.473545	-2.064948
5	1	H	-0.296676	1.252989	-1.787909
6	7	N	1.482802	0.170244	-1.993736
7	6	C	2.152520	-1.090719	-1.655999
8	1	H	3.188689	-1.032638	-1.985917
9	1	H	1.673543	-1.914146	-2.204100
10	6	C	2.050642	-1.367182	-0.144314
11	1	H	2.509147	-2.344816	0.032671
12	6	C	2.820311	-0.354758	0.711221
13	1	H	3.098291	-0.805408	1.667728
14	1	H	3.757394	-0.058100	0.237799
15	7	N	2.077096	0.890774	1.028078
16	6	C	2.862530	1.851375	1.740133
17	8	O	4.032480	1.594732	1.999103
18	6	C	2.168893	3.065454	2.097355
19	1	H	2.755962	3.810102	2.618280
20	6	C	0.847880	3.239554	1.815913
21	1	H	0.344637	4.152643	2.116810
22	6	C	0.116012	2.231306	1.139577
23	1	H	-0.940714	2.349392	0.937025
24	6	C	0.745593	1.074314	0.755846
25	6	C	0.578548	-1.388543	0.286809
26	1	H	0.021524	-2.152653	-0.264434
27	1	H	0.485183	-1.620933	1.351639
28	6	C	2.093112	1.111710	-2.734950
29	6	C	3.374167	1.090933	-3.511456
30	8	O	4.255719	0.281986	-3.672300
31	6	C	3.051638	2.534479	-3.932603
32	8	O	3.522558	3.400260	-4.631625
33	6	C	1.845164	2.440454	-3.087885
34	7	N	0.888756	3.373487	-2.844656
35	1	H	1.100192	4.298934	-3.194744
36	1	H	0.409203	3.366802	-1.955981

Table S1. (c) Optimized parameters for (−)-cytisine squaramide **2** dimeric structure (AB).

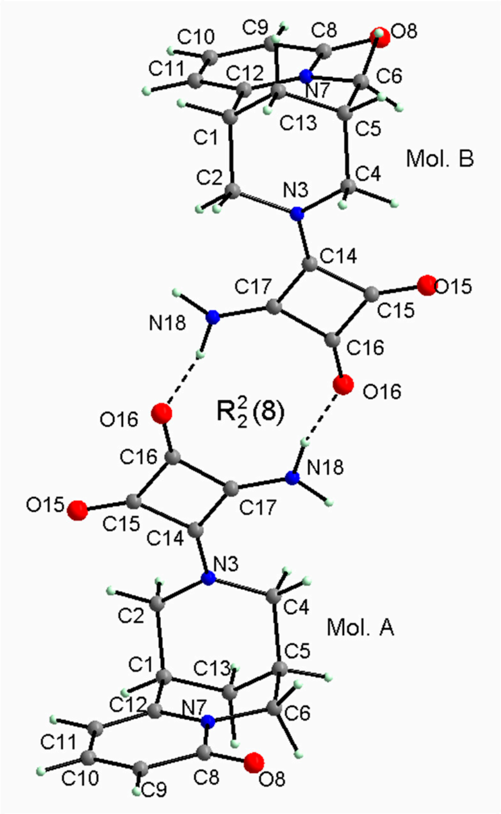

Hydrogen bond

D—H···*A*	*D*—H	H···*A*	*D*···*A*	*D*—H···A
N18A—H···O16	1.025	1.824	2.836	168.48
N18B—H···O16	1.024	1.827	2.838	168.49

Bonds (Å)					
C1–C2	1.544	C2–N3	1.468	N3–C4	1.464
C4–C5	1.550	C5–C6	1.531	C5–C13	1.532
C13–C1	1.533	C6–N7	1.480	N7–C8	1.428
C8–C9	1.441	C9–C10	1.363	C10–C11	1.416
C11–C12	1.369	C12–C1	1.515	C12–N7	1.374
C8–O8	1.228	N3–C14	1.345	C14–C15	1.488
C15–O15	1.213	C15–C16	1.517	C16–O16	1.221
C16–C17	1.472	C17–C14	1.412	C17–N18	1.335
C1–H	1.093	C4–H	1.095	C5–H	1.095
C6–H	1.093	C13–H	1.094	Car–H	1.083
N18–H	1.009/1.025				

Angles (°)					
C1–C2–N3	110.67	C2–N3–C4	115.45	N3–C4–C5	111.80
C4–C5–C6	113.41	C4–C5–C13	110.23	C5–C13–C1	106.61
C13–C1–C2	109.22	C5–C6–N7	114.81	C6–N7–C8	113.25
N7–C8–O8	119.15	N7–C8–C9	115.06	C8–C9–C10	121.44
C9–C10–C11	120.68	C10–C11–C12	119.78	C11–C12–C1	121.14
C11–C12–N7	119.83	C12–N7–C8	123.02	C2–N3–C14	119.52
C4–N3–C14	123.24	N3–C14–C15	131.26	C14–C15–O15	135.24
C14–C15–C16	87.32	C15–C16–O16	138.81	C15–C16–C17	86.62
C16–C17–N18	129.14	N18–C17–C14	138.10	C16–C17–C14	91.99

Torsion angles (°)					
C1–C2–N3–C4	51.88	C2–N3–C4–C5	-50.08	N3–C4–C5–C6	-68.73
N3–C4–C5–C13	54.16	C4–C5–C13–C1	-61.09	C5–C13–C1–C2	-62.93
C4–C5–C6–N7	83.61	C5–C6–N7–C8	-174.01	C6–N7–C8–O8	1.76
C6–N7–C8–C9	-177.97	N7–C8–C9–C10	2.37	C8–C9–C10–C11	-0.73
C9–C10–C11–C12	-0.81	C10–C11–C12–C1	-177.22	C10–C11–C12–N7	0.67
C11–C12–C1–C2	87.46	C12–N7–C8–O8	177.31	C1–C2–N3–C14	-142.84

C2–N3–C14–C15	10.61	N3–C14–C15–O15	-0.86	N3–C14–C15–C16	179.27
C14–C15–C16–O16	179.31	C14–C15–C16–C17	-0.35	O15–C15–C16–O16	-0.56
C15–C16–C17–N18	-179.79	C15–C16–C17–C14	0.36	O16–C16–C17–N18	0.53
O16–C16–C17–C14	179.31	N18–C17–C14–C15	179.81	C17–C14–C15–O15	-179.62
N18–C17–C14–N3	1.01	C17–C14–N3–C4	-6.90		

Direct from Gaussian

AB-dimer (OPTIMIZED)

Center Number	Atomic Number	Atomic Type	Coordinates (Angstroms) X	Y	Z
1	6	0	7.192017	1.586955	0.374092
2	1	0	7.892630	2.334760	-0.004820
3	6	0	5.772853	2.177094	0.225376
4	1	0	5.729578	3.124833	0.782548
5	1	0	5.531608	2.384991	-0.815471
6	7	0	4.767309	1.249399	0.758162
7	6	0	5.013663	0.768022	2.118419
8	1	0	4.303617	-0.025929	2.348046
9	1	0	4.851404	1.585309	2.836977
10	6	0	6.447566	0.231253	2.284206
11	1	0	6.571491	-0.003375	3.346057
12	6	0	6.704638	-1.066082	1.512400
13	1	0	7.527673	-1.617169	1.976540
14	1	0	5.843856	-1.736257	1.547254
15	7	0	7.056173	-0.881312	0.087194
16	6	0	7.155569	-2.110206	-0.632709
17	8	0	6.920077	-3.165809	-0.050140
18	6	0	7.541239	-1.978364	-2.014668
19	1	0	7.600564	-2.898454	-2.580715
20	6	0	7.831183	-0.763286	-2.558676
21	1	0	8.131321	-0.692311	-3.598787
22	6	0	7.746365	0.416072	-1.779322
23	1	0	7.969780	1.383833	-2.207913
24	6	0	7.353451	0.339197	-0.470087
25	6	0	7.464219	1.293855	1.854015
26	1	0	8.485762	0.931450	2.000680
27	1	0	7.351659	2.207695	2.445813
28	6	0	3.578991	1.132123	0.138164
29	6	0	3.104304	1.640094	-1.177292
30	8	0	3.589609	2.299411	-2.071721
31	6	0	1.777261	0.970251	-0.876838
32	8	0	0.694155	0.853820	-1.427872
33	6	0	2.341444	0.506065	0.400875
34	7	0	1.784314	-0.227219	1.367042
35	1	0	0.814977	-0.550374	1.288586
36	1	0	2.270614	-0.458695	2.215839
37	6	0	-6.676659	-0.230480	-1.901538
38	1	0	-6.855734	0.153886	-2.908762
39	6	0	-5.280477	-0.900111	-1.910262
40	1	0	-5.263180	-1.669148	-2.696813

41	1	0	-4.523380	-0.154074	-2.143948
42	7	0	-4.983238	-1.501002	-0.613992
43	6	0	-6.016970	-2.404882	-0.086564
44	1	0	-5.727829	-2.702391	0.920591
45	1	0	-6.049422	-3.311350	-0.708857
46	6	0	-7.402758	-1.740696	-0.102466
47	1	0	-8.118474	-2.502626	0.220867
48	6	0	-7.530941	-0.571016	0.879252
49	1	0	-8.577316	-0.434381	1.165648
50	1	0	-6.989368	-0.764531	1.806333
51	7	0	-7.057951	0.733770	0.358129
52	6	0	-7.106875	1.781531	1.328889
53	8	0	-7.493538	1.528540	2.465035
54	6	0	-6.687193	3.076768	0.852477
55	1	0	-6.694759	3.875171	1.582544
56	6	0	-6.330050	3.275851	-0.446521
57	1	0	-6.035240	4.264058	-0.783661
58	6	0	-6.344618	2.201757	-1.370255
59	1	0	-6.075542	2.355717	-2.407078
60	6	0	-6.702563	0.947602	-0.949973
61	6	0	-7.739435	-1.267680	-1.520549
62	1	0	-7.722480	-2.104679	-2.225778
63	1	0	-8.739244	-0.825787	-1.560902
64	6	0	-3.775533	-1.440914	-0.026362
65	6	0	-3.283245	-1.998298	1.262921
66	8	0	-3.767094	-2.667527	2.150017
67	6	0	-1.946391	-1.353073	0.948632
68	8	0	-0.851574	-1.266681	1.481746
69	6	0	-2.525976	-0.848205	-0.305808
70	7	0	-1.971588	-0.108120	-1.269676
71	1	0	-0.989581	0.177615	-1.211816
72	1	0	-2.463664	0.132377	-2.112525

Table S2. Optimized parameters for (−)-cytisine squaramide **3**.

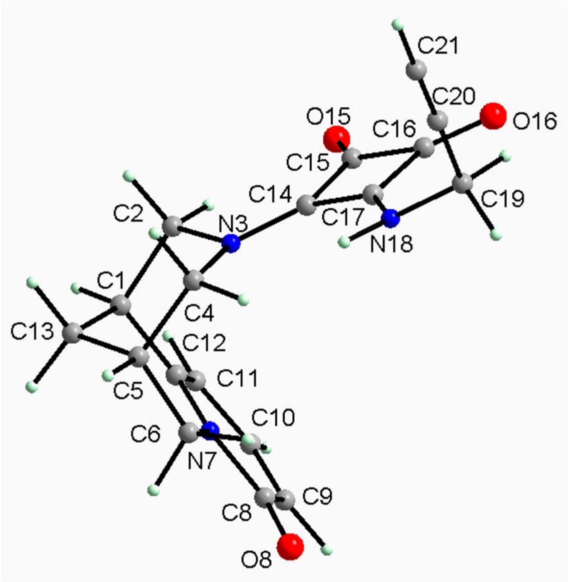

**Bonds (Å)**					
C1–C2	1.543	C2–N3	1.469	N3–C4	1.463
C4–C5	1.540	C5–C6	1.531	C5–C13	1.532
C13–C1	1.533	C6–N7	1.479	N7–C8	1.427
C8–C9	1.441	C8–O8	1.229	C9–C10	1.363
C10–C11	1.416	C11–C12	1.369	C12–N7	1.375
N3–C14	1.349	C14–C15	1.492	C15–O15	1.209
C15–C16	1.528	C16–O16	1.211	C16–C17	1.484
C17–C14	1.403	C17–N18	1.350	N18–C19	1.472
C19–C20	1.463	C20–C21	1.202	N18–H	1.009
C1–H	1.092	C2–H	1.101, 1.088	C4–H	1.101, 1.090
C5–H	1.095	C13–H	1.095	Car–H	1.083
C19–H	1.093	C21–H	1.063		
**Angles (°)**					
C1–C2–N3	110.72	C2–N3–C4	115.44	N3–C4–C5	111.86
C4–C5–C6	113.37	C4–C5–C13	110.31	C5–C13–C1	106.58
C13–C1–C2	109.20	C5–C6–N7	114.81	C6–N7–C8	113.28
N7–C8–O8	119.13	N7–C8–C9	115.07	C8–C9–C10	121.41
C9–C10–C11	120.80	C10–C11–C12	119.80	C11–C12–N7	119.34
C12–N7–C8	123.06	C2–N3–C14	119.14	N3–C14–C15	130.95
C14–C15–O15	135.00	C14–C15–C16	87.55	C15–C16–O16	137.69
C15–C16–C17	87.84	C16–C17–N18	130.50	C16–C17–C14	92.70
C17–C14–N3	136.91	C14–C17–N18	136.75	C17–N18–C19	121.53
N18–C19–C20	112.61	C19–C20–C21	178.76		
**Torsion angles (°)**					
C1–C2–N3–C4	-53.80	C2–N3–C4–C5	49.86	N3–C4–C5–C6	68.90
N3–C4–C5–C13	-54.36	C4–C5–C13–C1	61.02	C5–C13–C1–C2	-62.96
C4–C5–C6–N7	-83.46	C5–C6–N7–C8	173.48	C6–N7–C8–C9	177.45
C6–N7–C8–O8	-1.81	N7–C8–C9–C10	-2.65	C8–C9–C10–C11	0.72
C9–C10–C11–C12	0.76	C10–C11–C12–N7	-0.75	C11–C12–N7–C8	-0.76
C2–N3–C14–C15	-13.59	N3–C14–C15–O15	1.67	N3–C14–C15–C16	-178.10
C14–C15–C16–O16	179.22	C14–C15–C16–C17	-0.28	C15–C16–C17–N18	177.86
C15–C16–C17–C14	0.30	C16–C17–C14–N3	177.92	C16–C17–N18–C19	13.87
C17–N18–C19–C20	103.44	N18–C19–C20–C21	59.30		

Direct from Gaussian

(−)-cytisine squaramide **3**

Center Number	Atomic Number	Atomic Type	Coordinates (Angstroms) X	Y	Z
1	6	C	-0.108427	0.053599	-0.008800
2	1	H	0.582481	-0.064013	0.829250
3	6	C	-0.684980	1.482540	0.074427
4	1	H	-1.231393	1.639673	1.002386
5	1	H	0.150433	2.198484	0.045605
6	7	N	-1.594594	1.742199	-1.049043
7	6	C	-1.051816	1.453446	-2.376770
8	1	H	-0.289742	2.202081	-2.642728
9	1	H	-1.857396	1.515937	-3.108905
10	6	C	-0.421551	0.050317	-2.445798
11	1	H	0.048225	-0.031966	-3.431008
12	6	C	-1.448271	-1.081997	-2.350977
13	1	H	-2.362716	-0.847096	-2.898517
14	1	H	-1.047603	-1.986340	-2.818297
15	7	N	-1.846674	-1.444705	-0.973503
16	6	C	-2.908949	-2.396803	-0.925726
17	8	O	-3.405730	-2.792031	-1.977938
18	6	C	-3.296800	-2.812637	0.398000
19	1	H	-4.114281	-3.519098	0.453688
20	6	C	-2.651566	-2.349959	1.505287
21	1	H	-2.955293	-2.687166	2.490633
22	6	C	-1.582855	-1.428403	1.386972
23	1	H	-1.072243	-1.053477	2.263850
24	6	C	-1.201780	-0.983877	0.149484
25	6	C	0.628154	-0.115910	-1.342327
26	1	H	1.102815	-1.099252	-1.406990
27	1	H	1.417897	0.636377	-1.434285
28	6	C	-2.671349	2.530655	-0.853906
29	6	C	-3.306360	3.025500	0.402735
30	8	O	-3.067562	2.913518	1.583001
31	6	C	-4.357713	3.704880	-0.473764
32	8	O	-5.350913	4.379637	-0.320056
33	6	C	-3.636261	3.163226	-1.652259
34	7	N	-3.917576	3.252602	-2.970016
35	1	H	-3.229777	2.942284	-3.639772
36	6	C	-4.911583	4.209915	-3.481764
37	1	H	-5.553979	3.699505	-4.205821
38	1	H	-5.540167	4.515487	-2.642393
39	6	C	-4.295128	5.379092	-4.109908
40	6	C	-3.776881	6.323699	-4.642829
41	1	H	-3.327637	7.170246	-5.102569

Table S3. Optimized parameters for (−)-cytisine squaramide **4**.

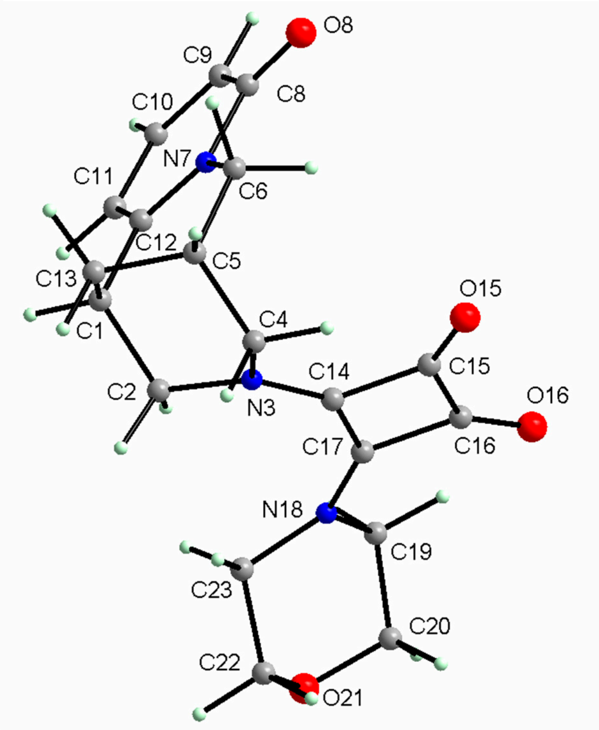

Bonds (Å)			
C1–C2	1.551	C2–N3	1.459
N3–C4	1.471	C4–C5	1.537
C5–C6	1.533	C6–N7	1.483
N7–C8	1.430	C8–O8	1.226
C8–C9	1.443	C9–C10	1.362
C10–C11	1.417	C11–C12	1.371
C12–N7	1.372	C12–C1	1.514
C1–C13	1.533	C13–C5	1.532
N3–C14	1.350	C14–C15	1.489
C15–O15	1.211	C15–C16	1.525
C16–O16	1.211	C16–C17	1.490
C17–C14	1.413	C17–N18	1.347
N118–C19	1.473	C19–C20	1.525
C20–O21	1.427	O21–C22	1.418
C22–C23	1.532	C23–N18	1.463
Car–H	1.083	C5–H	1.094
C4–H	1.089	C1–H	1.093
C2–H	1.094	C19–H	1.098
C20–H	1.095	C23–H	1.098
C22–H	1.095		

Angles (°)					
C1–C2–N3	111.29	C2–N3–C4	115.65	N3–C4–C5	112.06
C4–C5–C6	113.37	C4–C5–C13	109.80	C5–C13–C1	106.58
C13–C1–C2	109.84	C5–C6–N7	114.81	C6–N7–C8	113.27
N7–C8–O8	119.27	N7–C8–C9	115.07	C8–C9–C10	121.49
C9–C10–C11	120.56	C10–C11–C12	119.78	C11–C12–N7	120.20
C12–N7–C8	122.80	C2–N3–C14	124.32	N3–C14–C15	129.52
C14–C15–O15	134.82	C14–C15–C16	87.81	C15–C16–O16	137.44
C15–C16–C17	87.81	C16–C17–N18	129.47	C16–C17–C14	92.08
C17–C14–N3	138.30	C14–C17–B18	138.32	C17–N18–C19	120.47
N18–C19–C20	110.40	C19–C20–O21	111.15	C20–O21–C22	111.15
O21–C22–C23	111.50	C22–C23–N18	109.84	C23–N18–C19	114.55

Torsion angles (°)					
C1–C2–N3–C4	49.73	C2–N3–C4–C5	-49.69	N3–C4–C5–C6	-68.12
C1–C2–N3–C14	-128.14	C2–N3–C14–C15	158.30	C2–C1–C12–C11	88.49
N3–C4–C5–C13	55.39	C4–C5–C13–C1	-61.71	C5–C13–C1–C2	62.20
C6–C5–C13–C1	63.74	C4–C5–C6–N7	86.20	C5–C6–N7–C8	-177.82
C6–N7–C8–C9	-179.03	C6–N7–C8–O8	0.61	N7–C8–C9–C10	3.16
C8–C9–C10–C11	-0.89	C9–C10–C11–C12	-1.07	C10–C11–C12–N7	0.51
C11–C12–N7–C8	2.05	C2–N3–C14–C15	159.30	N3–C14–C15–O15	6.25
N3–C14–C15–C16	-175.47	C14–C15–C16–O16	176.25	C14–C15–C16–C17	-2.78
C15–C16–C17–N18	-175.26	C15–C16–C17–C14	2.93	C16–C17–C14–N3	174.14
O16–C16–C17–N18	5.66	C16–C17–N18–C19	-19.78	C17–N18–C19–C20	126.42
N18–C19–C20–O21	53.39	C19–C20–O21–C22	-60.20	C20–O21–C22–C23	60.67
O21–C22–C23–N18	-54.18	C22–C23–N18–C17	-126.27	C23–N18–C17–C15	-21.50
C23–N18–C17–C16	155.77	N18–C17–C14–N3	-6.97	N18–C17–C14–C15	174.89

Direct from Gaussian

(−)-cytisine squaramide **4**

Center Number	Atomic Number	Atomic Type	Coordinates (Angstroms) X	Y	Z
1	6	C	0.026626	-0.071201	0.008183
2	1	H	0.104471	0.837324	0.611040
3	6	C	1.402891	-0.276657	-0.675649
4	1	H	1.651449	0.641001	-1.230039
5	1	H	2.164514	-0.436458	0.084483
6	7	N	1.380402	-1.422931	-1.577466
7	6	C	0.261686	-1.460606	-2.531396
8	1	H	0.281702	-2.421506	-3.043156
9	1	H	0.413194	-0.669757	-3.280603
10	6	C	-1.093648	-1.237466	-1.840907
11	1	H	-1.845275	-1.187869	-2.634753
12	6	C	-1.503289	-2.391225	-0.917753
13	1	H	-2.592143	-2.434965	-0.831610
14	1	H	-1.192738	-3.355592	-1.323141
15	7	N	-0.971739	-2.301677	0.463825
16	6	C	-1.332601	-3.418064	1.281504
17	8	O	-2.007585	-4.320480	0.799690
18	6	C	-0.853483	-3.360137	2.641494
19	1	H	-1.093361	-4.211529	3.264477
20	6	C	-0.167838	-2.280276	3.108873
21	1	H	0.162661	-2.252374	4.142029
22	6	C	0.112466	-1.181948	2.258986
23	1	H	0.638428	-0.311496	2.628962
24	6	C	-0.290306	-1.214618	0.949185
25	6	C	-1.065943	0.077626	-1.056154
26	1	H	-0.840846	0.921805	-1.716154
27	1	H	-2.033730	0.278169	-0.587330
28	6	C	2.281188	-2.428593	-1.553090
29	6	C	2.163173	-3.819106	-2.072570
30	8	O	1.334920	-4.421573	-2.718681
31	6	C	3.537548	-4.097275	-1.473080
32	8	O	4.281489	-5.044196	-1.349682
33	6	C	3.584031	-2.666269	-1.060472
34	7	N	4.583474	-1.974653	-0.479643
35	6	C	5.667598	-2.679072	0.225415
36	1	H	5.534623	-3.752544	0.095162
37	1	H	5.602391	-2.437703	1.294179
38	6	C	7.024796	-2.239576	-0.312379
39	1	H	7.829107	-2.641644	0.305175
40	1	H	7.155718	-2.599199	-1.342584
41	8	O	7.157486	-0.819079	-0.276191
42	6	C	6.160142	-0.191659	-1.065494
43	1	H	6.257814	-0.506124	-2.115332
44	1	H	6.339707	0.883438	-1.002543
45	6	C	4.752727	-0.523714	-0.559713
46	1	H	4.607857	-0.071704	0.430757
47	1	H	4.017436	-0.107725	-1.247607

Table S4. Optimized parameters for (−)-cytisine squaramide **5**.

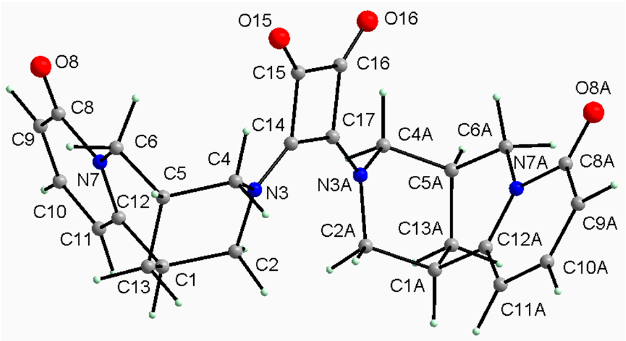

Bonds (Å)					
C1–C2	1.549	C2–N3	1.457	N3–C4	1.472
C4–C5	1.537	C5–C6	1.533	C6–N7	1.483
N7–C8	1.430	C8–O8	1.226	C8–C9	1.443
C9–C10	1.362	C10–C11	1.417	C11–C12	1.371
C12–N7	1.371	C12–C1	1.515	C1–C13	1.532
C13–C5	1.531	N3–C14	1.351	C14–C15	1.491
C15–O15	1.210	C15–C16	1.525	C16–O16	1.210
C16–C17	1.491	C17–C14	1.413	N3A–C17	1.351
C1A–C2A	1.549	C2A–N3A	1.457	N3A–C4A	1.472
C4A–C5A	1.537	C5A–C6A	1.533	C6A–N7A	1.483
N7A–C8A	1.430	C8A–O8A	1.226	C8A–C9A	1.443
C9A–C10A	1.362	C10A–C11A	1.417	C11A–C12A	1.371
C12A–N7A	1.371	C12A–C1A	1.515	C1A–C13A	1.532
C13A–C5A	1.531				
Angles (°)					
C1–C2–N3	111.36	C2–N3–C4	115.69	N3–C4–C5	112.05
C4–C5–C6	113.47	C4–C5–C13	109.85	C5–C13–C1	106.47
C13–C1–C2	109.94	C5–C6–N7	114.82	C6–N7–C8	113.29
N7–C8–O8	119.27	N7–C8–C9	115.07	C8–C9–C10	121.49
C9–C10–C11	120.57	C10–C11–C12	119.77	C11–C12–N7	120.19
C12–N7–C8	122.83	C2–N3–C14	124.43	N3–C14–C15	128.82
C14–C15–O15	134.78	C14–C15–C16	87.83	C15–C16–O16	137.38
C15–C16–C17	87.83	C16–C17–N3A	128.82	C16–C17–C14	92.12
C1A–C2A–N3A	111.36	C2A–N3A–C4A	115.69	N3A–C4A–C5A	112.05
C4A–C5A–C6A	113.47	C4A–C5A–C13A	109.85	C5A–C13A–C1A	106.47
C13A–C1A–C2A	109.94	C5A–C6A–N7A	114.82	C6A–N7A–C8A	113.29
N7A–C8A–O8A	119.27	N7A–C8A–C9A	115.07	C8A–C9A–C10A	121.49
C9A–C10A–C11A	120.57	C10A–C11A–C12A	119.77	C11A–C12A–N7A	120.19
Torsion angles (°)					
C1–C2–N3–C4	49.50	C2–N3–C4–C5	-49.48	N3–C4–C5–C6	-68.23
N3–C4–C5–C13	55.37	C4–C5–C13–C1	-61.75	C5–C13–C1–C2	62.18
C6–C5–C13–C1	63.85	C4–C5–C6–N7	-86.28	C5–C6–N7–C8	-177.78
C6–N7–C8–C9	-179.00	C6–N7–C8–O8	0.65	N7–C8–C9–C10	3.02
C8–C9–C10–C11	-0.82	C9–C10–C11–C12	-1.07	C10–C11–C12–N7	0.52
C11–C12–N7–C8	1.95	C2–N3–C14–C15	161.01	N3–C14–C15–O15	5.89
N3–C14–C15–C16	-175.31	C14–C15–C16–O16	176.37	C14–C15–C16–C17	-2.37
C15–C16–C17–N3a	-175.31	C15–C16–C17–C14	2.50	C16–C17–C14–N3	174.84
C1A–C2A–N3A–C4A	49.50	C2A–N3A–CA4–C5A	-49.48	N3A–C4A–C5A–C6A	-68.23
N3A–C4A–CA5–C13A	55.37	C4A–C5A–C13A–C1A	-61.75	C5A–C13A–C1A–C2A	62.18
C6A–C5A–C13A–C1A	63.85	C4A–C5A–C6A–N7A	-86.26	C5A–C6A–N7A–C8A	-177.76
C6A–N7A–C8A–C9A	-179.00	C6A–N7A–C8A–O8A	0.65	N7A–C8A–C9A–C10A	-3.02
C8A–C9A–C10A–C11A	-0.83	C9A–C10A–C11A–C12A	-1.07	C10A–C11A–C12A–N7A	0.52
C11A–C12A–N7A–C8A	1.95	C2A—N3A–C17–C16	161.00	N3A–C17–C16–O16	5.89

Direct from Gaussian

(−)-cytisine squaramide **4**

Center Number	Atomic Number	Atomic Type	Coordinates (Angstroms) X	Y	Z
1	6	C	0.532986	0.546501	2.224155
2	8	O	1.130643	1.207746	3.042178
3	6	C	-0.533489	-0.543233	2.224770
4	8	O	-1.131338	-1.203350	3.043574
5	6	C	-0.515796	-0.482318	0.735374
6	6	C	0.515638	0.483539	0.734810
7	6	C	2.982309	0.782737	-1.943057
8	1	H	3.029186	0.536624	-3.007106
9	6	C	1.487565	0.735524	-1.537839
10	1	H	0.927920	1.402678	-2.211856
11	1	H	1.114375	-0.278005	-1.668043
12	7	N	1.301098	1.136206	-0.149516
13	6	C	1.959245	2.391743	0.246041
14	1	H	1.863176	2.501473	1.325157
15	1	H	1.432385	3.231530	-0.231148
16	6	C	3.435815	2.427847	-0.179022
17	1	H	3.807294	3.426468	0.070381
18	6	C	4.315003	1.428835	0.582511
19	1	H	5.347798	1.785630	0.614525
20	1	H	3.997618	1.328894	1.621690
21	7	N	4.355449	0.065464	0.000510
22	6	C	5.143628	-0.851893	0.763657
23	8	O	5.685826	-0.464498	1.792220
24	6	C	5.227786	-2.185660	0.219154
25	1	H	5.797884	-2.898783	0.799641
26	6	C	4.644894	-2.506994	-0.968910
27	1	H	4.741662	-3.511899	-1.366777
28	6	C	3.918626	-1.535627	-1.701178
29	1	H	3.472275	-1.777439	-2.656974
30	6	C	3.782439	-0.266881	-1.199987
31	6	C	3.549201	2.182946	-1.686470
32	1	H	4.589271	2.243802	-2.019804
33	1	H	2.980593	2.931904	-2.247270
34	6	C	-2.982178	-0.785113	-1.942286
35	1	H	-3.029066	-0.540226	-3.006619
36	6	C	-1.487446	-0.737291	-1.537062
37	1	H	-0.927718	-1.405238	-2.210231
38	1	H	-1.114315	0.276099	-1.668498
39	7	N	-1.301050	-1.136225	-0.148234
40	6	C	-1.959133	-2.391288	0.248906
41	1	H	-1.863240	-2.499529	1.328190
42	1	H	-1.432094	-3.231669	-0.227040
43	6	C	-3.435636	-2.428184	-0.176345
44	1	H	-3.806943	-3.426585	0.074205
45	6	C	-4.315203	-1.428481	0.583813
46	1	H	-5.347991	-1.785316	0.615685
47	1	H	-3.998311	-1.327487	1.623040
48	7	N	-4.355419	-0.065693	0.000448
49	6	C	-5.143647	0.852498	0.762499

50	8	O	-5.686128	0.466202	1.791331
51	6	C	-5.227497	2.185763	0.216696
52	1	H	-5.797492	2.899562	0.796452
53	6	C	-4.644546	2.505779	-0.971690
54	1	H	-4.741128	3.510291	-1.370597
55	6	C	-3.918413	1.533540	-1.702942
56	1	H	-3.472069	1.774287	-2.659012
57	6	C	-3.782360	0.265317	-1.200410
58	6	C	-3.548892	-2.185101	-1.684098
59	1	H	-4.588923	-2.246531	-2.017450
60	1	H	-2.980106	-2.934631	-2.243956

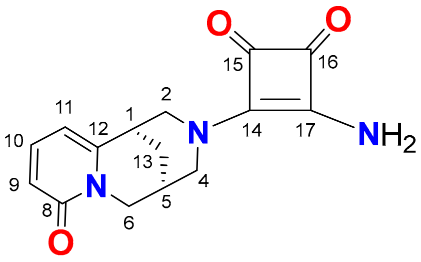

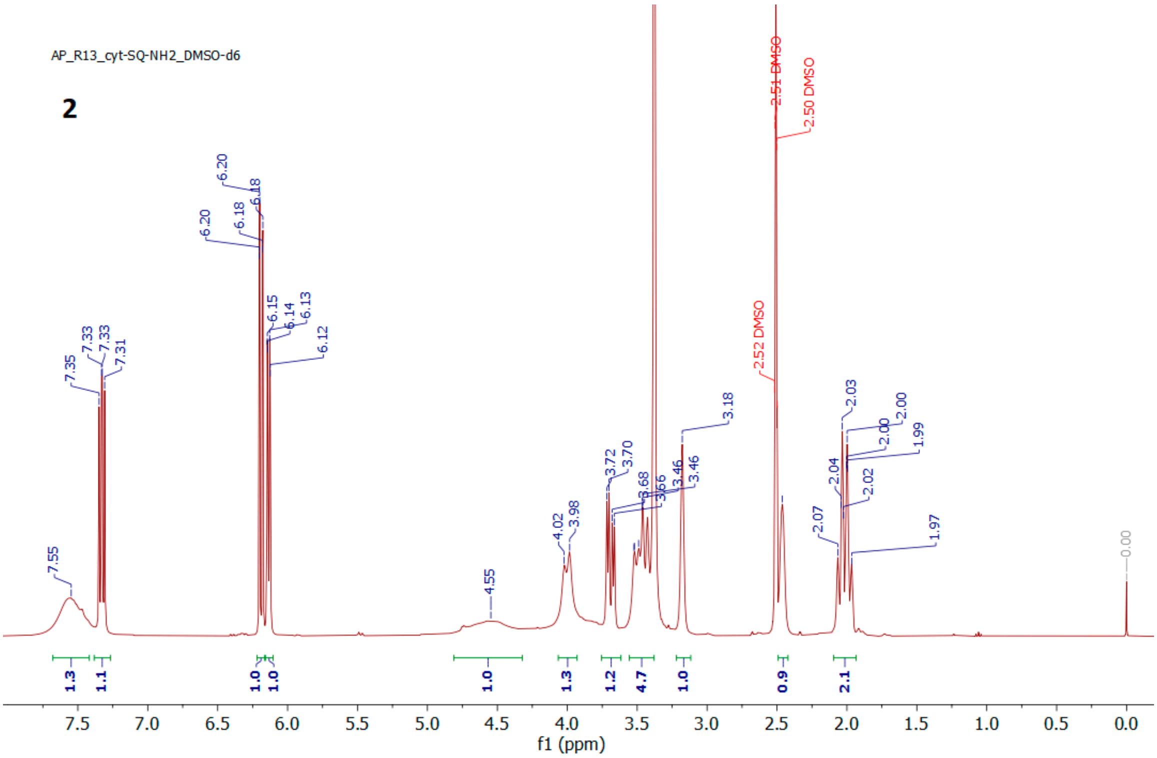

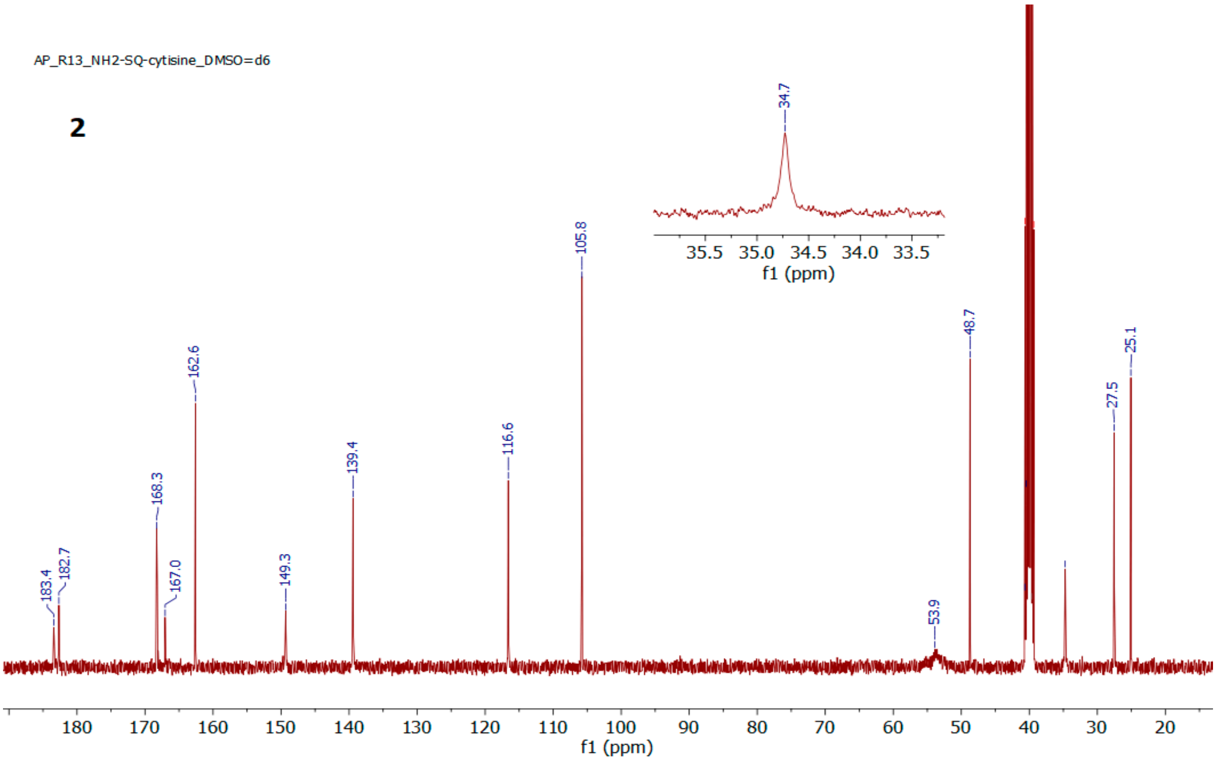

Figure S1. ^{1}H-NMR (600 MHz) and ^{13}C-NMR (150 MHz) spectra of (−)-cytisine squaramide **2** in DMSO-d$_6$.

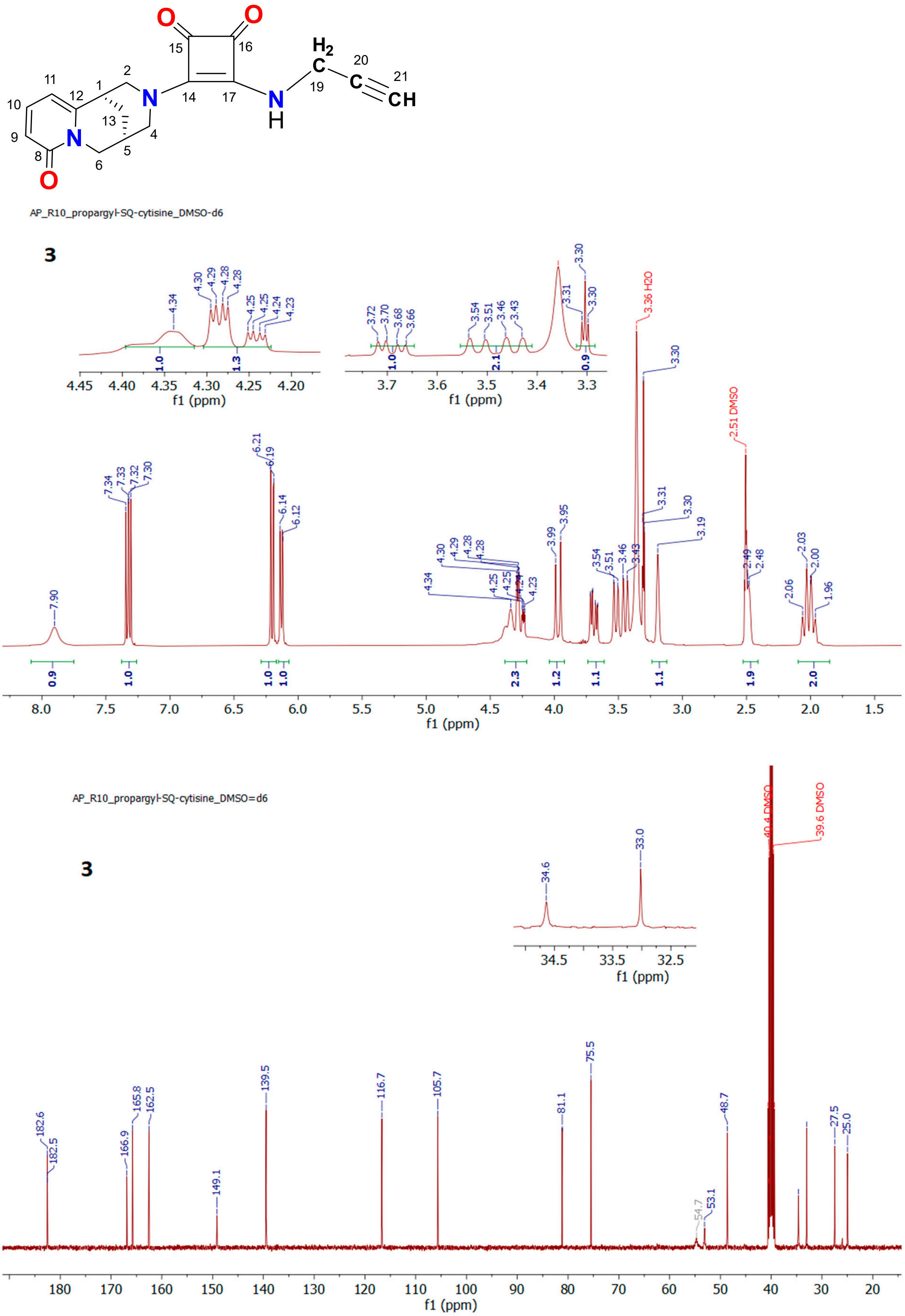

Figure S2. ^{1}H-NMR (600 MHz) and ^{13}C-NMR (150 MHz) spectra of (−)-cytisine squaramide **3** in DMSO-d_6.

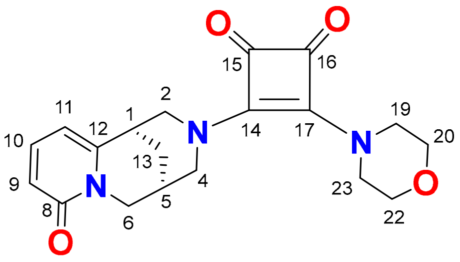

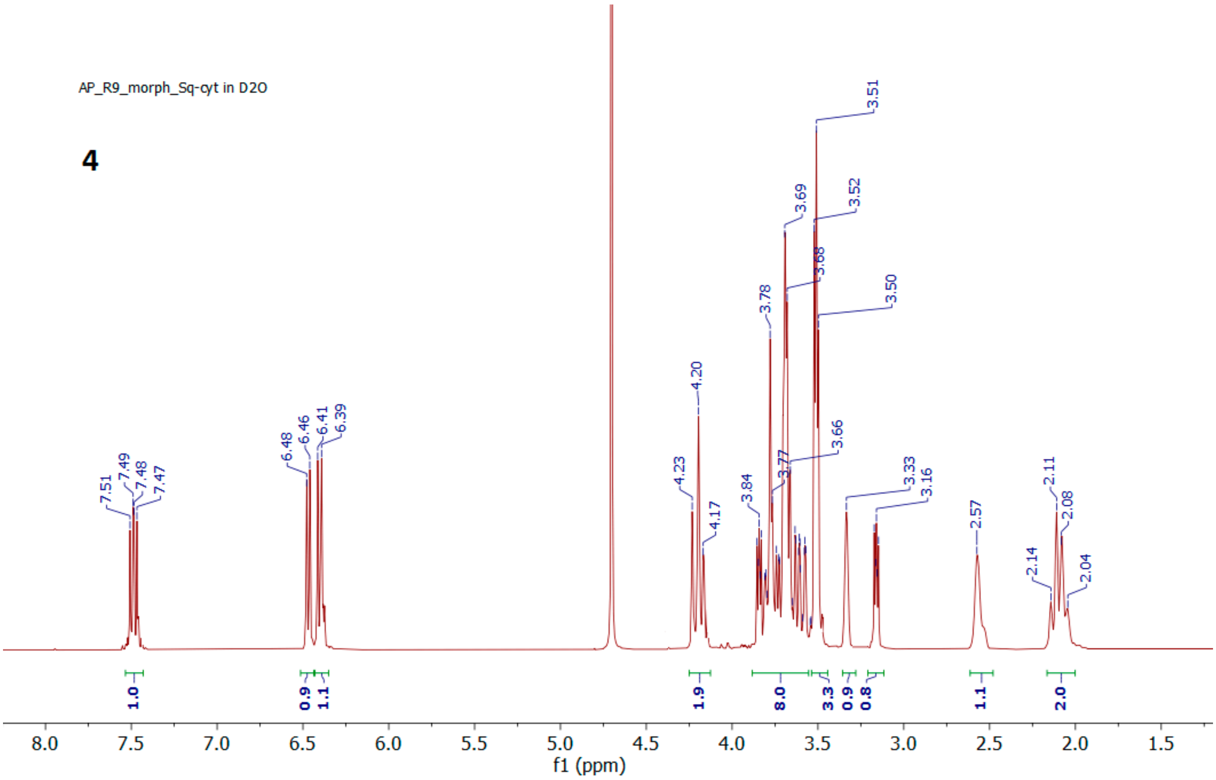

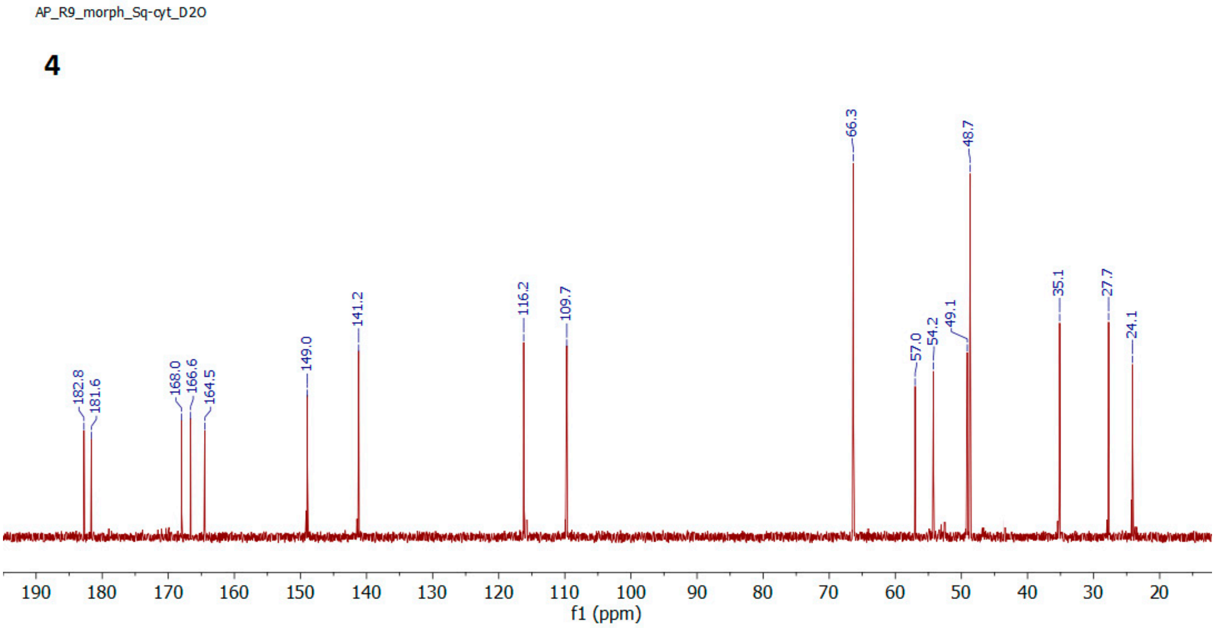

Figure S3. ^{1}H-NMR (600 MHz) and ^{13}C-NMR (150 MHz) spectra of (−)-cytisine squaramide **4** in D_2O.

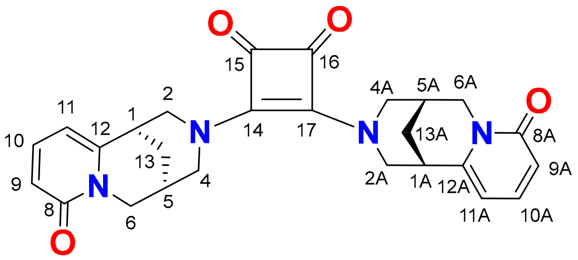

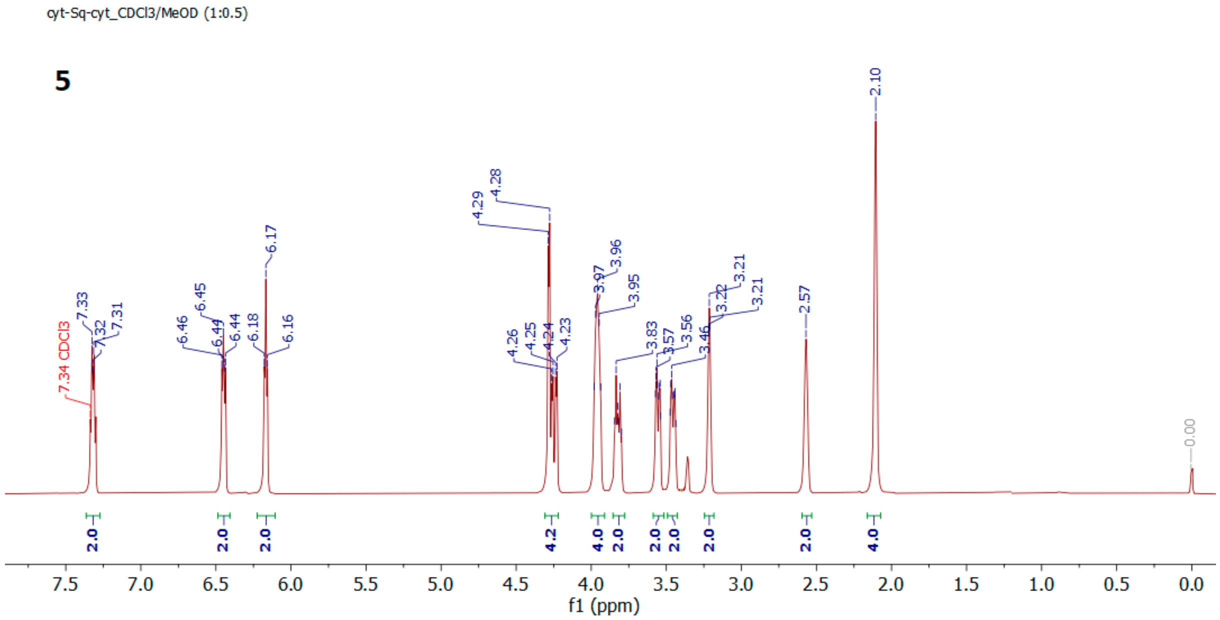

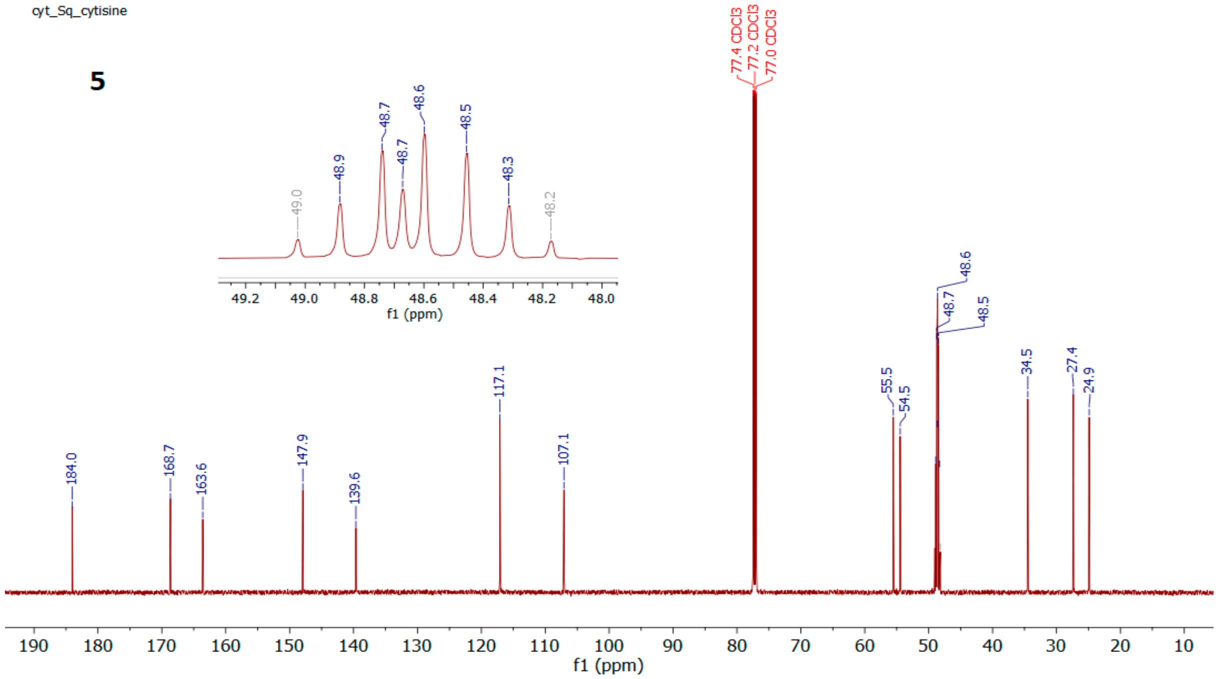

Figure S4. ^{1}H-NMR (600 MHz) and ^{13}C-NMR (150 MHz) spectra of (−)-cytisine squaramide **5** in $CDCl_3$:CD_3OD (1:0.5).
